# Supplementary material for: A metabolic atlas of the Klebsiella pneumoniae species complex reveals lineage-specific metabolism and capacity for intra-species co-operation
Source: PLoS Biol. 2025 Dec 12;23(12):e3003559. doi: 10.1371/journal.pbio.3003559 (PMC12700438; doi:10.1371/journal.pbio.3003559)
Supplement: S1 Text — (DOCX) [file pbio.3003559.s001.docx]

Supplementary Text

# A metabolic atlas of the *Klebsiella pneumoniae* species complex reveals lineage-specific metabolism and capacity for intra-species co-operation

Ben Vezina^1,2*^, Helena B. Cooper^1,2^, Christopher K. Barlow^3^, Martin Rethoret-Pasty^4^, Sylvain Brisse^4^, Jonathan M. Monk^5^, Kathryn E. Holt^1,6^ and Kelly L. Wyres^1,2,6*^

^1^ Department of Infectious Diseases, School of Translational Medicine, Monash University, Melbourne, Victoria, Australia

^2^ Centre to Impact AMR, Monash University, Clayton, Victoria, Australia

^3^ Monash Proteomics and Metabolomics Platform, Department of Biochemistry and Molecular Biology, Biomedicine Discovery Institute, Monash University, Victoria 3800, Australia

^4^ Institut Pasteur, Université Paris Cité, Biodiversity and Epidemiology of Bacterial Pathogens, Paris, France

^5^ Department of Medicine, University of California, San Diego School of Medicine, San Diego, CA, United States of America

^6^ Department of Infection Biology, London School of Hygiene and Tropical Medicine, London, UK

* Corresponding authors Ben Vezina ([benjamin.vezina@monash.edu](mailto:benjamin.vezina@monash.edu)); Kelly L. Wyres ([Kelly.wyres@monash.edu](mailto:Kelly.wyres@monash.edu))

## Total number of substrates predicted to support growth varies by strain

Individual *Kp*SC differed in terms of the total number of substrates predicted to support growth, with a general trend towards fewer substrates supported in anaerobic compared to aerobic conditions (262-379 each for aerobic vs 4-320 anaerobic). There was greater variability among usage of carbon sources (104-210 aerobic, 2-173 anaerobic) compared to those used as sources of nitrogen (87-109 aerobic, 2-91 anaerobic), phosphorous (36-52 aerobic, 0-52 anaerobic), and sulfur (7-14 aerobic, 0-10 anaerobic) (**Fig. S1A**). This likely reflects a genuine biological trend underpinned by greater variability of carbon metabolic processes as well as biases in our underlying knowledge about metabolism (i.e. carbon metabolism is the most well understood). There were 17 outlier strains which produced predicted growth in only four substrates under anerobic conditions. These were made up of 13 *K. pneumoniae*, 2 *K. quasipneumoniae subsp. similipneumoniae* and 2 *K. variicola subsp. tropica* of various sub-lineages, but were all from the same study (1). This is likely a DNA sequencing artefact as the Nextera XT library preparation was used in this study, which can cause coverage bias issues (2) and likely contributed to Bactabolize not being able to find some key metabolic genes.

## Substrate usage predictions support species differentiation

As expected, our growth predictions suggested that *Kp*SC taxa are differentiated by core substrate usage patterns (**Fig. S2, S5 Data**). Four taxa were represented by sufficient genomes for comparisons (n>200 each); *K. pneumoniae* (hereafter *Kp,* n=6,652), *Klebsiella quasipneumoniae* subsp. *quasipneumoniae* (*Kqq*, n=201), *K. quasipneumoniae* subsp. *similipneumoniae* (*Kqs*, n=285) and *Klebsiella variicola* subsp. *variicola* (*Kvv,* n=672). Among these taxa, a total of 52 distinct growth conditions (corresponding to 18 distinct substrates) were core to at least one but variable and/or absent (not predicted to support growth of any isolates) from at least one other taxon. This included 31 growth conditions that were uniquely core to a single taxon (range 0-16).

All taxa appeared to dedicate similar proportions of their genomes to metabolic orthologs (51.3-54%, **S1 Data**), but there were statistically significant differences in the proportions assigned to key macromolecular functions as determined by analysis of Clusters of Orthologous Gene (COG) categories (**Fig. S4**). Notably, *Kp* appeared to have a larger portion of its genes involved with carbohydrate metabolism (median 13.2% vs 12.6-12.9%, p < 0.0001 for all comparisons) compared to the other well sampled taxa, and a smaller portion in amino acid transport and metabolism (median 10.04% vs 10.45-10.56%, p < 0.0001 for all comparisons). *Kvv* has a smaller portion involved in nucleotide transport and metabolism (median 2.38% vs 2.5-2.53%, p < 0.0001), and a larger portion in amino acid metabolism (median 10.56% vs 10.04-10.49%, p < 0.0001 for all comparisons).

When compared to the biochemical tests used for formal species definitions, the growth predictions were generally consistent. However, there were several cases that we defined as minor discrepancies where biochemical testing indicated complete absence or conservation of a capability within the KpSC or a specific taxon, and our predictions indicated <5% or >95% conservation, respectively (**S7 Data**). These differences likely reflect rare strain variations that have been captured by our genome collection but were not present among the biochemical test data. Seventeen larger-scale discrepancies were also identified; two may reflect inaccuracies in the model predictions for L-carnitine usage, which we previously estimated at ≤30% accuracy (3). Three discrepancies were associated with D-lactic acid methyl ester for which we currently have no prediction accuracy estimate, but for which the biochemically-derived and predicted conservation levels were consistent for taxa represented by ≥200 genomes each. The final 12 discrepancies were associated with substrates for which we have previously confirmed high predictive accuracy from metabolic models (≥94.6%, **S6 Data**). It is most likely that these discrepancies were driven by under-sampling in the biochemical testing set, resulting in inaccurate conservation estimates and indicating that the formal species definitions should be revisited.

## Species and sub-lineages show unique metabolic ortholog fingerprints

Predicted growth phenotype variability is driven by variation in gene and reaction content in the metabolic models, but these represent only the subset of true metabolic genes for which the relevant reaction stoichiometries and supporting literature evidence is available for inclusion in the reference model (3). Therefore, we also explored the distribution of the broader set of metabolic orthologs which showed that each taxon was associated with a distinct core metabolic profile (**Fig. S3, S4 Data**). Individual taxa harboured between 145 and 207 core metabolic orthologs beyond the 1,375 that were core to the species complex as a whole. Among the four well sampled taxa (n>200) 63 metabolic orthologs were uniquely core to a single taxon (and absent or variably present in other taxa), whereas 40 were core to at least two taxa and absent or variably present in the others (**Fig. S3,** **S4 Data**).

Within *Kp*, the 48 common sub-lineages were associated with 1,489-1,592 core metabolic orthologs each, plus 75-416 variable orthologs (**S4 Data**). Overall, 51 metabolic orthologs were majority sub-lineage specific core (core to ≥66% of sub-lineages); 56 orthologs were common sub-lineages specific core (≥25% to <66% of sub-lineages) and 127 were rare sub-lineage specific core. 888 metabolic orthologs were not core to any sub-lineage but variably present in between 0 and 48 well-represented sub-lineages.

## Co-occurrence analysis of metabolic traits

Co-occurrence analysis was performed on growth phenotype predictions to identify genetically linked and/or co-selected traits. Isolates were subsampled to a maximum of 10 genomes per sub-lineage to control for population sampling biases (n=2,427 genomes, **S1 Data**). Twelve pairs of growth phenotypes (substrate usage) were detected as co-occurring in this dataset including five instances of a single substrate utilised as alternative element sources (e.g. carbon and nitrogen) plus four additional mechanistically linked substrate pairs: Methanesulfonate (as a sulfur source) and methanol usage were linked by their converging degradation into formaldehyde, though methanesulfonate additionally produces sulfite as a sulfur source. Galactitol and D-tagatose usage were linked by their common degradation into D-tagatofuranose 1,6-bisphosphate by two distinct enzymes (tagatose-bisphosphate aldolase and phosphofructokinase). Fructoselysine and xylitol usage were linked by their converging degradation into D-ribulose 5-phosphate, a key substrate for the pentose phosphate pathway and an essential substrate for biomass production. Acetate and butyrate are closely linked by their direct degradation into butanoyl-CoA, an important co-enzyme involved in fatty acid metabolism. In contrast, for the twelfth pair formamide and Fe(III)dicitrate, we could not identify a common mechanistic pathway or enzyme, indicating co-occurrence may be due to co-selection or physical genetic linkage.

## Experimental validation of growth predictions

*In vitro* growth assays were performed on seven substrates as sole sources of carbon plus allantoin as a sole source of nitrogen and methanesulfonate as a sole source of sulfur in m9 minimal media, aerobic atmosphere. These were selected to represent common and rare sub-lineage specific core growth capabilities that had not been validated previously (3) as well as two positive controls that are known to support growth of all *K. pneumoniae* (glucose and glycerol). Two experiments were performed, i) measuring OD_600_ at 24- and 48-hour endpoints; ii) a substrate coaxing experiment (see **Methods**). This second experiment was performed for substrates where one or more metabolic model predicted growth but growth was not observed in the initial 48-hour culture assays. These types of false positive model predictions are most likely due to regulatory mechanisms that are not considered by the models and may indicate that the isolates were not expressing relevant genes. To address this, isolates were coaxed onto the testing substrate via subculturing into progressively lower amounts of glycerol (core carbon source; 10 mM on subculture 1, 5 mM on subculture 2, 0 mM on subculture 3) plus the substrate of interest.

A total of 13 distinct *K. pneumoniae* isolates, representing nine distinct sub-lineages, were tested in triplicate, grown aerobically on each of the nine substrates (**S2 Data**)**.** As expected, all isolates were able to grow in M9 plus glucose and M9 plus glycerol. Similarly, all isolates that were predicted to grow in M9 plus L-hydroxyproline were able to do so, as were two additional isolates (AJ229, INF269) that were not predicted to be able to utilise this substrate. Unexpectedly, acetoacetate, allantoin and methanesulfonate supported growth of 12/13, 13/13 and 12/13, isolates respectively, including 10, 11 and nine isolates that were not predicted to grow. These results indicate that the metabolic models are missing genes and reactions that support the metabolism of these substrates, but database and literature searches did not resolve these issues.

Growth on allantoin as a nitrogen source was particularly interesting as it has been previously shown to be specific and in-fact essential for virulence in *K. pneumoniae* SL23 (4). Further investigation showed that the necessary reactions to support allantoin metabolism were present in all models, but the transport reaction, required for transport of the substrate into the cytosol, was missing in most. The exceptions were models carrying the *all* accessory operon including those from SL23 (**Fig. 3**). However, our *in vitro* coaxing experiments indicate that all *K. pneumoniae* may be able to utilise this substrate to some degree given the right conditions. As our experiments grew to three days at 60 mM allantoin (considerably higher concentration than tested previously (4)), we were able to detect this growth. We predict that SL23 may utilise allantoin more efficiently, due to the presence of the dedicated transport machinery, but further investigations are needed (beyond the scope of this work).

In contrast to those discussed above, we were not able to demonstrate growth of any isolates on butyrate or methanol as sole sources of carbon. We suspected that we did not capture the correct growth conditions for these substrates, as the genes required for their metabolism were clearly present within some of the test isolate genomes. Notably, previously generated growth profiles of 37 isolates in aerobic (5) and anaerobic conditions (3), indicated that *Kp*SC prefer anerobic usage of butyrate; 17/37 isolates were able to grow on butyrate resulting in a model predictive accuracy of 94.59%.

Finally, the *in vitro* data indicated several inaccuracies for prediction of growth on xylitol, with three false positive and four false negative model predictions for the 13 tested isolates.

## Identification of putative cross-feeding metabolites

In order to better understand the interactions between the auxotroph and prototroph isolate pairs tested *in vitro*, we simulated co-cultures *in silico* using MICOM for each of one representative pair (6). This approach aims to optimise growth of the community (isolate pairs in this case) and will therefore favour mutualistic interactions. Since we had already demonstrated such interactions *in vitro*, we were seeking to identify the set of putative cross-feeding metabolites, i.e. those associated with reaction fluxes indicating export from the prototroph and uptake by the auxotroph. Between 20 and 29 putative cross-feeding metabolites were predicted for each pair, totalling 44 unique metabolites (**S8 Data**). Next we sought to confirm the presence of these metabolites in the supernatants of the representative prototrophs using metabolomics analyses, which were expected to be able to detect 35 of the 44 predicted cross-feeding metabolites (the remaining nine were below the minimum detectable mass, **S8 Data**). Notably, 28 of the predicted cross-feeding metabolites were included within the standards library and therefore identified with high confidence. The full list of putative metabolites detected, median peak intensities for the isolate supernatants and matched control samples, log_2_ fold change and statistical analysis outcomes are listed in **S8 Data**.

Several notable predicted cross-feeding metabolites known to support ubiquitous growth of KpSC were associated with substantial peak intensity increases in isolate supernatants compared to the matched controls (labelled in **Fig. S8**). We used a literature search to identify the associated exporter proteins in *E. coli* (alanine, AlaE, NP_417156.1; glutamate, MscS, NP_417399.1; malate, DcuA, CitT, and TtdT, NP_418561.1, NP_415145.1 and NP_417535.1; lactate, SetA, YP_025293.1; pyruvate, PykF, NP_416191.1) or *Tetragenococcus halophila* (aspartate, Asp, BAB92081.1). tBLASTn confirmed that putative orthologs were present in all of the representative prototroph genomes (≥89% coverage and ≥49% identity, with the exception of the aspartate exporter which was found at ~27% identity in all genomes.

# Additional references

1. Huynh B-T, Passet V, Rakotondrasoa A, Diallo T, Kerleguer A, Hennart M, et al. *Klebsiella pneumoniae* carriage in low-income countries: antimicrobial resistance, genomic diversity and risk factors. Gut Microbes. 2020;11(5):1287-99.

2. Gunasekera S, Abraham S, Stegger M, Pang S, Wang P, Sahibzada S, et al. Evaluating coverage bias in next-generation sequencing of Escherichia coli. PLoS One. 2021;16(6):e0253440.

3. Cooper HB, Vezina B, Hawkey J, Passet V, López-Fernández S, Monk JM, et al. A validated pangenome-scale metabolic model for the *Klebsiella pneumoniae* species complex. Microbial Genomics. 2024;10(2).

4. Chou HC, Lee CZ, Ma LC, Fang CT, Chang SC, Wang JT. Isolation of a chromosomal region of Klebsiella pneumoniae associated with allantoin metabolism and liver infection. Infect Immun. 2004;72(7):3783-92.

5. Blin C, Passet V, Touchon M, Rocha EPC, Brisse S. Metabolic diversity of the emerging pathogenic lineages of *Klebsiella pneumoniae*. Environmental Microbiology. 2017;19(5):1881-98.

6. Diener C, Gibbons Sean M, Resendis-Antonio O. MICOM: Metagenome-Scale Modeling To Infer Metabolic Interactions in the Gut Microbiota. mSystems. 2020;5(1):e00606-19.

7. Imhoff J. Bergey’s Manual of Systematic Bacteriology. 2005. p. 587-850.

8. Rodrigues C, Passet V, Rakotondrasoa A, Diallo TA, Criscuolo A, Brisse S. Description of *Klebsiella africanensis* sp. nov., *Klebsiella variicola* subsp. *tropicalensis* subsp. nov. and *Klebsiella variicola* subsp. *variicola*subsp. nov. Res Microbiol. 2019;170(3):165-70.

9. Wyres KL, Lam MMC, Holt KE. Population genomics of *Klebsiella pneumoniae*. Nature Reviews Microbiology. 2020;18(6):344-59.
