# Supplementary material for: A metabolic atlas of the Klebsiella pneumoniae species complex reveals lineage-specific metabolism and capacity for intra-species co-operation
Source: PLoS Biol. 2025 Dec 12;23(12):e3003559. doi: 10.1371/journal.pbio.3003559 (PMC12700438; doi:10.1371/journal.pbio.3003559)
Supplement: S7 Data — (DOCX) [file pbio.3003559.s008.docx]

**S7 Data:** Biochemical tests described in formal species definitions and consistency with model predictions.

| **Biochemical test** | **Taxa (phylogroup)** | | | | | | | **Growth prediction**  **accuracy (%) (3)** |
| --- | --- | --- | --- | --- | --- | --- | --- | --- |
|  | *Kp*  (Kp1) | *Kqq*  (Kp2) | *Kvv*  (Kp3) | *Kqs*  (Kp4) | *Kvt*  (Kp5) | *Kqv*  (Kp6) | *Ka*  (Kp7) |  |
| **Substrates for which growth was thought to be conserved among KpSC (7)** | | | | | | | | |
| D-Glucose | (100) | (100) | (100) | (100) | (100) | (100) | (100) | 100 |
| L-Arabinose | (100) | (100) | (100) | (100) | (100) | (100) | (100) | 100 |
| D-Mannitol | (100) | (100) | (100) | (100) | (100) | (100) | (100) | 92 |
| D-Mannose | (100) | (100) | (100) | (98) | (100) | (100) | (100) | 100 |
| Melibiose | (100) | (100) | (100) | (98) | **(60)** | (100) | (100) | 100 |
| Raffinose | (100) | (100) | (100) | (100) | (100) | (100) | (100) | 100 |
| L-Rhamnose | (100) | (100) | (100) | (100) | (100) | (100) | (100) | 97 |
| Salicin | (100) | (100) | (100) | (100) | (100) | (100) | (100) | 100 |
| D-Sorbitol | (100) | (100) | (100) | (98) | (100) | (100) | (100) | 100 |
| Sucrose | (99) | (100) | (100) | (99) | (100) | (100) | (100) | 100 |
| Trehalose | (100) | (100) | (100) | (100) | (100) | (100) | (100) | 100 |
| D-Xylose | (100) | (100) | (100) | (100) | (100) | (100) | (100) | 78 |
| D-Arabitol | (99) | (100) | (99) | (100) | (100) | (100) | **(0)** | 97 |
| Cellobiose | (100) | (100) | (100) | (100) | (100) | (100) | (100) | 100 |
| Lactose | (100) | (100) | (100) | (100) | (100) | (100) | (100) | 100 |
| Maltose | (97) | (100) | (100) | (98) | (100) | (100) | **(0)** | 95 |
| Glycerol | (100) | (100) | (100) | (100) | (100) | (100) | (100) | 97 |
| Urea (nitrogen) | (100) | (100) | (100) | (100) | (100) | (100) | (100) | Not tested |
| L-Tartrate | **(67)** | **(74)** | (100) | **(30)** | (100) | (100) | (100) | 95 |
| myo-Inositol | (100) | (100) | (100) | (98) | (100) | (100) | (100) | 100 |
| **Substrates that differentiate KpSC taxa (8)** |  |  |  |  |  |  |  |  |
| Dulcitol | ~ (48) | - (1) | ~ (58) | ~ (34) | ~ (80) | + (100) | + (100) | 100.0 |
| Adonitol | + (99) | ~ (63) | - (1) | + (99) | - (0) | - (0) | - (0) | 100.0 |
| Tricarballylic acid | - (0) | + (100) | ~ (100) | + (97) | + (60) | ~ (93) | + (100) | 94.6 |
| N-acetyl-neuraminic acid | - (0) | - (0) | - (0) | + (97) | - (0) | - (0) | - (0) | 100.0 |
| D-arabitol | + (99) | + (100) | + (99) | + (100) | + (100) | + (100) | - (0) | 100.0 |
| L-sorbose | ~ (53) | - (33) | + (100) | - (0) | + (100) | + (100) | + (100) | 97.3 |
| D-tagatose | ~ (48) | - (1) | ~ (58) | ~ (34) | ~ (80) | + (100) | + (100) | 100.0 |
| 5-keto-D-gluconic acid | - (0) | ~ (41) | + (100) | - (3) | + (100) | + (100) | + (100) | 100.0 |
| D-lactic acid methyl ester | + (100) | + (100) | + (100) | + (99) | - (100) | - (100) | - (100) | Not tested |
| 4-hydroxyl-L-proline | - (11) | + (75) | + (100) | + (98) | + (100) | + (100) | + (29) | 100.0 |
| L-carnitine | ~ (0) | - (0) | + (0) | + (0) | + (0) | - (0) | + (0) | 29.7 |
| **Sample sizes** |  |  |  |  |  |  |  |  |
| N isolates (biochemical tests) (8) | 10 | 5 | 5 | 4 | 6 | 5 | 1 | N/A |
| N genomes (growth predictions) (This study) | 6652 | 201 | 672 | 285 | 5 | 13 | 7 | N/A |

Substrate usage conservation as determined by biochemical testing reported in (7, 8) and predicted in this study (parentheses) is shown for each KpSC taxon: Kp = *K. pneumoniae,* Kqq = K. *quasipneumoniae* subsp. *quasipneumoniae*, Kvv = *K. variicola* subsp. *variicola,* Kqs = K. *quasipneumoniae* subsp. *similipneumoniae*, Kvt = *K. variicola* subsp. *tropica*, Kqv = *K. quasivariicola,* Ka = *K. africana* (labels in parentheses are phylogroup designations reported in [Imhoff J. Bergey’s Manual® of Systematic Bacteriology. 2005. p. 587-850]. ‘+’ indicates 100% conservation in biochemical testing, ‘-’ indicates 0% conservation in biochemical testing, ‘~’ indicates >0% and <100% conservation in biochemical testing (absolute values not reported). Values in parentheses indicate the percentage of isolates predicted to be able to utilise the substrate in this work. Formatting indicates the level of concordance between the conservation values derived from biochemical tests and those derived from growth predictions; none = conservation levels are consistent; underline = minor discrepancies i.e. predicted conservation level <5% different from biochemical conservation level (applied only where biochemical testing indicated 0% or 100% conservation); bold and grey shading = conservation levels are not consistent. Substrate level growth prediction accuracies are also indicated, as reported in [Cooper HB, Vezina B, Hawkey J, Passet V, López-Fernández S, Monk JM, et al. A validated pangenome-scale metabolic model for the *Klebsiella pneumoniae* species complex. Microbial Genomics. 2024;10(2).].
