# Supplementary material for: A metabolic atlas of the Klebsiella pneumoniae species complex reveals lineage-specific metabolism and capacity for intra-species co-operation
Source: PLoS Biol. 2025 Dec 12;23(12):e3003559. doi: 10.1371/journal.pbio.3003559 (PMC12700438; doi:10.1371/journal.pbio.3003559)
Supplement: S8 Data — (DOCX) [file pbio.3003559.s009.docx]

**S8 Data:** Substrates predicted to be involved in cross-feeding and LC-MS detection

| **Substrate (GSMMs)** | **Metabolite (LC-MS)** | **INF171** | **INF225** | **INF354** | **AJ155** | **INF120** | **INF359** |
| --- | --- | --- | --- | --- | --- | --- | --- |
| (R)-Glycerate | Glyceric acid φ |  |  | 5.4682  FC 11.12 * | 9.5754 FC 2.04 * |  |  |
| 2 Hydroxybutyrate | 2-Hydroxybutyric acid |  | 0.0003  FC 1.74 * |  |  |  | 0.0000  FC 1.03 * |
| 2-Oxoglutarate | Oxoglutaric acid φ |  | 6.0711  FC 8.51 * | 0.7533  FC 3.62 * | 1.2338  FC 7.78 * | 2.1210  FC 8.12 * | 1.9025  FC 8.50 * |
| Adenine | Adenine φ | 0.0000  FC 2.21 * |  |  |  |  |  |
| D-Alanine | Alanine φ | 0.0000  FC 0.09 |  |  | 0.0000  FC 5.76 * | 0.0000  FC 6.16 * | 0.0000  FC 7.50 * |
| D-Fructuronate | Fructuronate |  |  |  | 0.0038 FC na |  |  |
| D-lactate | Lactic acid φ | 0.7728  FC 0.02 | 0.3417  FC 6.53 * |  | 3.5006  FC 3.49 * | 4.4331  FC 3.33 * | 4.1051  FC 4.62 * |
| Fumarate | Fumaric acid φ | 0.9046  FC 0.33 |  |  |  |  |  |
| Glycerol | Glycerol φ | 1.0660  1.14 |  | 0.0272  FC 0.01 | 0.7781  FC 0.51 * | 0.6302  FC 0.6 * | 0.6788  FC 0.53 * |
| Glycine | Glycine φ | 0.0006  FC -0.02 | 0.0007  FC 1.75 * |  |  | 0.0461  FC 0.79 * | 0.0460  FC 1.58 * |
| Indole | Indole φ |  | 0.0002  FC 0.35 | 0.0000  FC 0.20 |  | 0.0257  FC -0.02 | 0.0255  FC 0.12 * |
| Inosine | Inosine φ |  |  |  |  | 0.1905  FC 8.11 | 0.1900  FC 8.11 |
| L-Arginine | Arginine φ |  |  | 0.0000  FC 0.23 | 0.0011 FC -0.31 |  | 0.0002  FC 0.56 |
| L-Aspartate | Aspartic acid φ | 0.2705  FC 0.18 | 1.4993  FC 2.22 * | 0.1837  FC 1.10 | 0.1018  FC 5.18 * |  |  |
| L-Glutamate | Glutamic acid φ | 2.3087  FC 1.73 * |  | 0.1127  FC 2.47 * | 0.8843  FC 7.96 * | 0.7855  FC 9.59 * | 0.7417  FC 8.11 * |
| L-Histidine | Histidine φ |  | 0.0006  FC 0.95 |  |  | 0.0461  FC -0.16 | 0.0461  FC 0.18 |
| L-Lactate | Lactic acid φ | 1.0406  FC 0.024 | 0.3182  FC 6.53 * |  | 3.5355  FC 3.49 * | 4.6058  FC 3.33 * | 4.1189  FC 4.62 * |
| L-Malate | Malic acid φ |  | 0.8014  FC 7.57 * |  | 2.2597  FC 5.04 * | 3.0263  FC 4.61 * | 2.8969  FC 5.64 * |
| L-Phenylalanine | Phenylalanine φ | 0.0000  FC 0.19 | 0.0000  FC 0.63 |  |  | 0.0002  FC 0.19 |  |
| L-Proline | Proline φ |  |  |  | 0.0000  FC 1.27 * | 0.0002  FC 1.46 * |  |
| L-Serine | Serine φ |  | 0.0776  FC -0.12 | 0.0003  FC -0.02 |  | 0.6934  FC -0.55 | 0.6933  FC -0.61 |
| L-Threonine | Threonine φ | 0.0002  FC 0.34 | 0.0005  FC 2.86 * | 0.0004  FC 1.13 * |  | 0.0003 FC 3.05 * | 0.0006  FC 2.91 * |
| L-Tyrosine | Tyrosine φ |  | 0.0105  FC 1.01 | 0.0001  FC 0.64 |  | 0.1432  FC 1.67 * | 0.1437  FC 1.51 * |
| L-Valine | Valine φ | 0.0019  FC 0.16 |  | 0.0013  FC -0.01 |  | 0.0006  FC 3.02 * |  |
| Pyruvate | Pyruvic acid φ |  | 7.6905  FC 6.57 * | 0.2657  FC 0.56 * | 2.1897  FC 2.16 * | 3.5267  FC 1.98 * | 3.1049  FC 2.74 * |
| 4-aminobenzoyl-glutamate | None detected | 3.3969 |  |  |  |  |  |
| Carbamyl Phosphate | None detected | 0.0000 |  | 0.0002 | 0.3057 |  |  |
| Guanosine | None detected |  |  |  |  | 0.1902 | 0.1907 |
| L-Asparagine | None detected φ | 0.0002 |  | 0.0000 | 0.0000 | 0.0003 | 0.0002 |
| L-Isoleucine | None detected φ | 0.0001 |  |  | 0.0002 | 0.0000 | 0.0000 |
| L-Leucine | None detected φ | 0.0007 | 0.0002 | 0.0000 | 0.0000 |  | 0.0003 |
| L-Tryptophan | None detected φ |  | 0.0000 |  |  | 0.0084 | 0.0087 |
| Ornithine | None detected φ | 0.0005 | 0.0004 |  |  | 0.0002 |  |
| Acetaldehyde | - |  | 18.3305 | 3.7821 | 11.8407 | 6.4294 | 5.9603 |
| Acetate | - | 2.2487 |  |  |  |  |  |
| Ammonia | - | 0.8282 |  |  |  |  |  |
| CO2 | - | 10.7410 | 17.2613 | 17.2314 | 14.9580 | 10.0444 | 9.0004 |
| D-Glyceraldehyde | - |  | 3.5863 |  |  |  |  |
| Dihydroxyacetone | - |  | 14.5222 | 0.0000 | 0.0019 |  |  |
| Ethanol | - | 2.6702 |  |  | 5.9583 | 4.0605 | 3.6794 |
| Formate | - | 1.8288 |  | 4.4253 |  |  |  |
| H+ | - | 7.1716 | 5.0816 |  | 19.2593 | 19.7411 | 18.3206 |
| H2 | - |  | 24.8360 |  | 28.5349 | 14.1342 | 10.1785 |
| H2O | - | 7.6805 | 8.0716 | 11.0418 | 12.3009 | 21.4053 | 23.4766 |

Substrates predicted to be involved with cross-feeding are shown by name as indicated in genome-scale metabolic models (GSMMs) and by the corresponding metabolite name provided in LC-MS outputs. φ indicates metabolite identification supported by comparison to a library standard, whereas other identifications are putative. Metabolites that can be detected by our LC-MS approach, but which were not detected in any samples are indicated as ‘None detected’. Metabolites that cannot be detected by our LC-MS approach are indicated by ‘-‘. Columns labelled INFx and AJ155 indicate the predicted flux for the corresponding substrate export from the named prototroph isolates (predicted by MICOM, empty cells indicate that the substrate was not predicted to be involved with cross-feeding). Where flux is predicted and the metabolite was measured by LC-MS we provide the fold-change (FC) in median peak intensity detected by LC-MS (culture supernatant vs no isolate control), * indicates significant difference (p<0.05 by Wilcox Rank Sum test with Benjamini Hochberg multiple testing correction). FC na indicates metabolite was not detected for this isolate nor its matched control. Note, that in this analysis it is not possible to resolve enantiomers and so the measurement will correspond to the sum of both.
