# Supplementary material for: A metabolic atlas of the Klebsiella pneumoniae species complex reveals lineage-specific metabolism and capacity for intra-species co-operation
Source: PLoS Biol. 2025 Dec 12;23(12):e3003559. doi: 10.1371/journal.pbio.3003559 (PMC12700438; doi:10.1371/journal.pbio.3003559)
Supplement: S1 Fig — (PDF) [file pbio.3003559.s010.pdf]

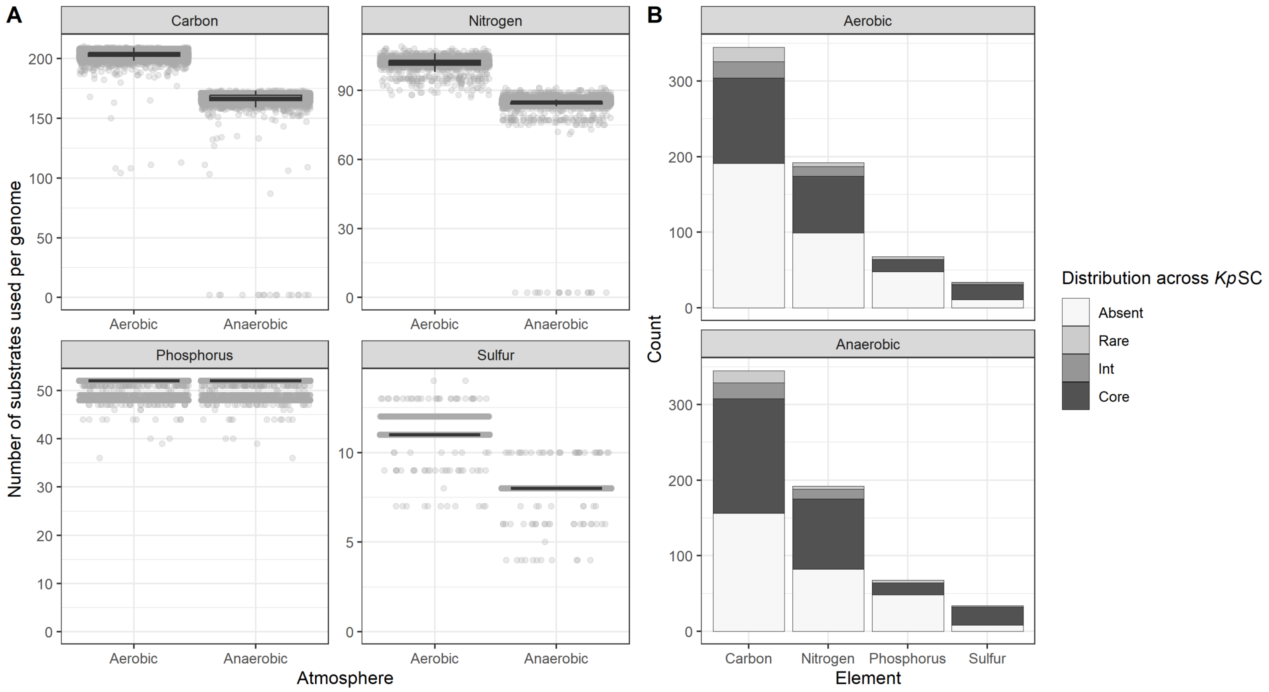

Fig. S1: Substrate usage frequencies across *Kp*SC dataset

A: Number of substrates predicted to support growth for each isolate, stratified by substrate type and atmosphere. **B:** Population frequency of predicted substrate usage by substrate type. Core refers to substrate usage conserved in ≥95% of isolates. 'Int' refers to intermediately present (>15% to <95%). Rare refers to >0 to <15%, while 'Absent' indicates no predicted growth. The data underlying this Figure can be found in **S5 Data**.
