# Supplementary material for: A metabolic atlas of the Klebsiella pneumoniae species complex reveals lineage-specific metabolism and capacity for intra-species co-operation
Source: PLoS Biol. 2025 Dec 12;23(12):e3003559. doi: 10.1371/journal.pbio.3003559 (PMC12700438; doi:10.1371/journal.pbio.3003559)
Supplement: S2 Fig — (PDF) [file pbio.3003559.s011.pdf]

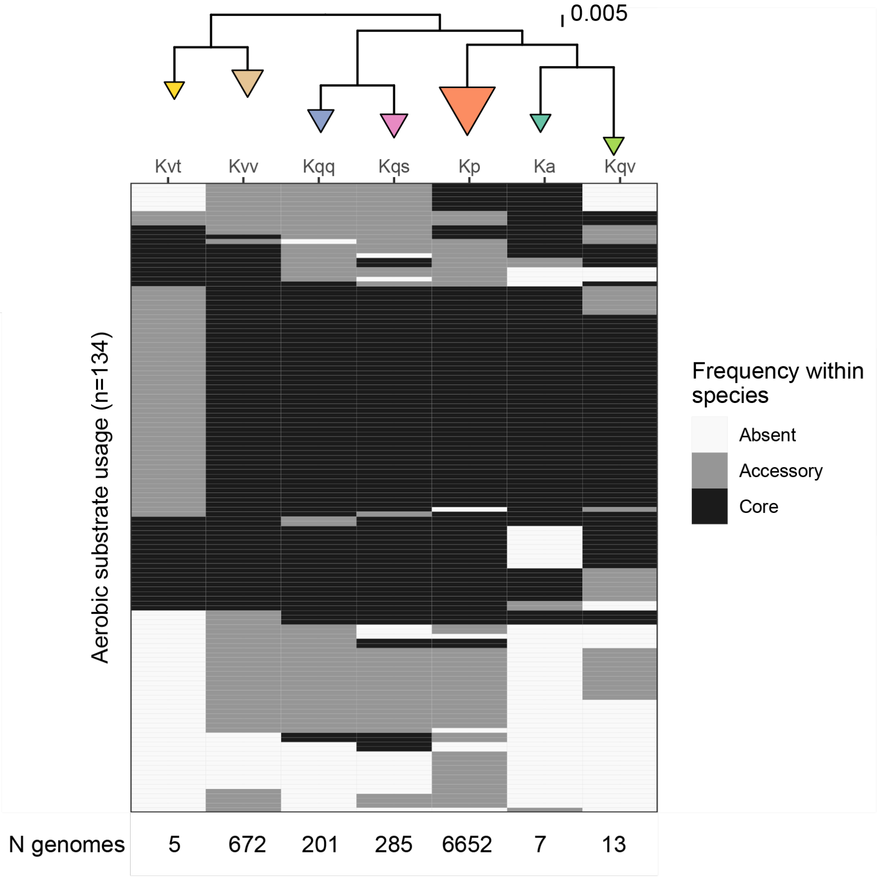

Fig. S2: Taxon-specific substrate usage

Heatmap showing taxon-specific substrate usage as predicted using metabolic models. Only substrates for which usage frequencies differed in aerobic conditions are shown. Columns are arranged by species-phylogeny and the number of genomes per taxon is shown along the bottom of the x-axis and arrowhead size. Taxa names shortened for brevity: Ka = *K. africana*. Kp = *K. pneumoniae*. Kqq = K. *quasipneumoniae* subsp. *quasipneumoniae*. Kqs = K. *quasipneumoniae* subsp. *similipneumoniae*. Kqv = *K. quasivariicola*. Kvt = *K. variicola* subsp. *tropica*. Kvv = *K. variicola* subsp. *variicola*. Tree file available from https://dx.doi.org/10.6084/m9.figshare.24503737. The data underlying this Figure can be found in **S5 Data**.
