# Supplementary material for: A metabolic atlas of the Klebsiella pneumoniae species complex reveals lineage-specific metabolism and capacity for intra-species co-operation
Source: PLoS Biol. 2025 Dec 12;23(12):e3003559. doi: 10.1371/journal.pbio.3003559 (PMC12700438; doi:10.1371/journal.pbio.3003559)
Supplement: S3 Fig — (PDF) [file pbio.3003559.s012.pdf]

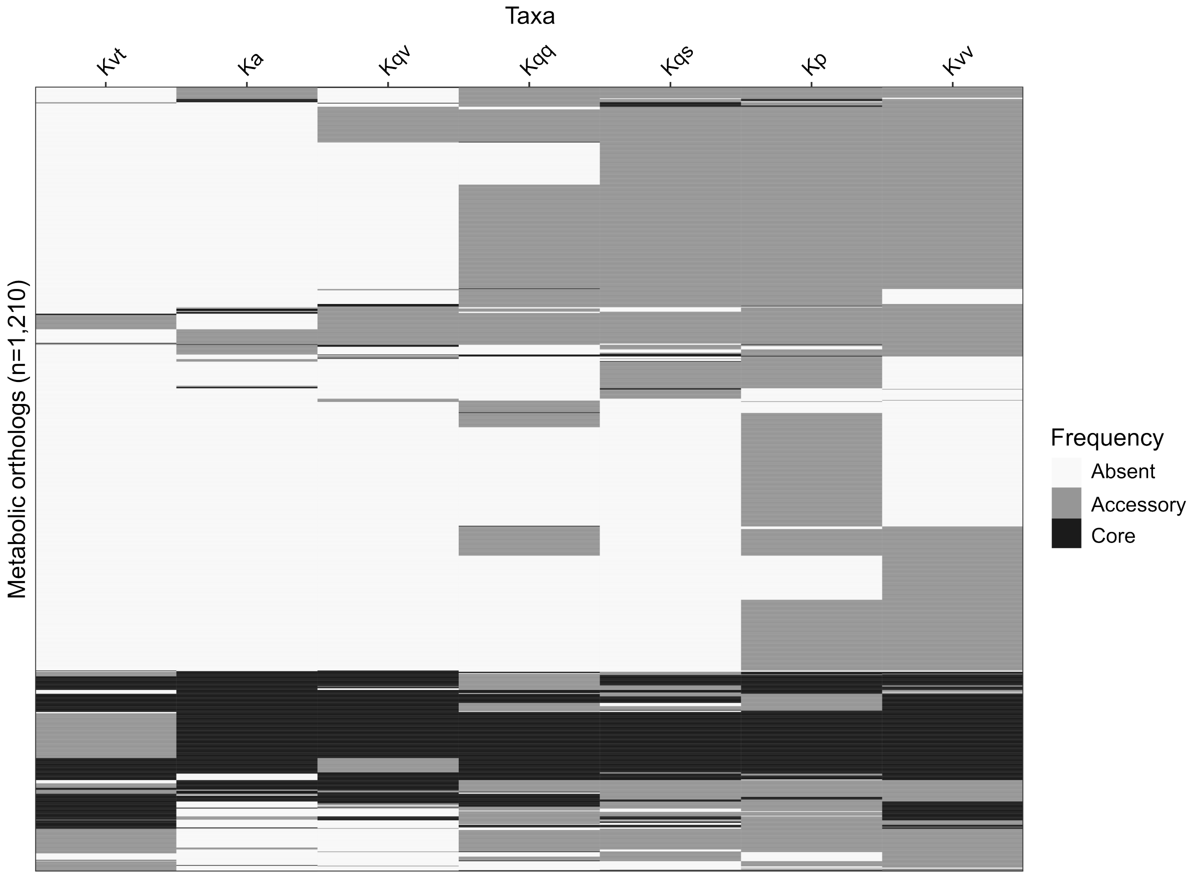

Fig. S3: Taxa are associated with unique metabolic ortholog profiles

Heatmap showing taxon-specific metabolic orthologs. Only orthologs for which frequencies differed between taxa are shown. Columns are arranged by species-phylogeny as in **Fig. S2** and names shortened for brevity: Ka = *K. africana*. Kp = *K. pneumoniae*. Kqq = K. *quasipneumoniae* subsp. *quasipneumoniae*. Kqs = K. *quasipneumoniae* subsp. *similipneumoniae*. Kqv = *K. quasivariicola*. Kvt = *K. variicola* subsp. *tropica*. Kvv = *K. variicola* subsp. *variicola*. Frequencies are indicated by colours as shown in the legend: Absent = not present in any genomes; Accessory = present in >0% and <95% genomes; Core = present in ≥95% genomes. The data underlying this Figure can be found in **S4 Data**.
