# Supplementary material for: A metabolic atlas of the Klebsiella pneumoniae species complex reveals lineage-specific metabolism and capacity for intra-species co-operation
Source: PLoS Biol. 2025 Dec 12;23(12):e3003559. doi: 10.1371/journal.pbio.3003559 (PMC12700438; doi:10.1371/journal.pbio.3003559)
Supplement: S4 Fig — (PDF) [file pbio.3003559.s013.pdf]

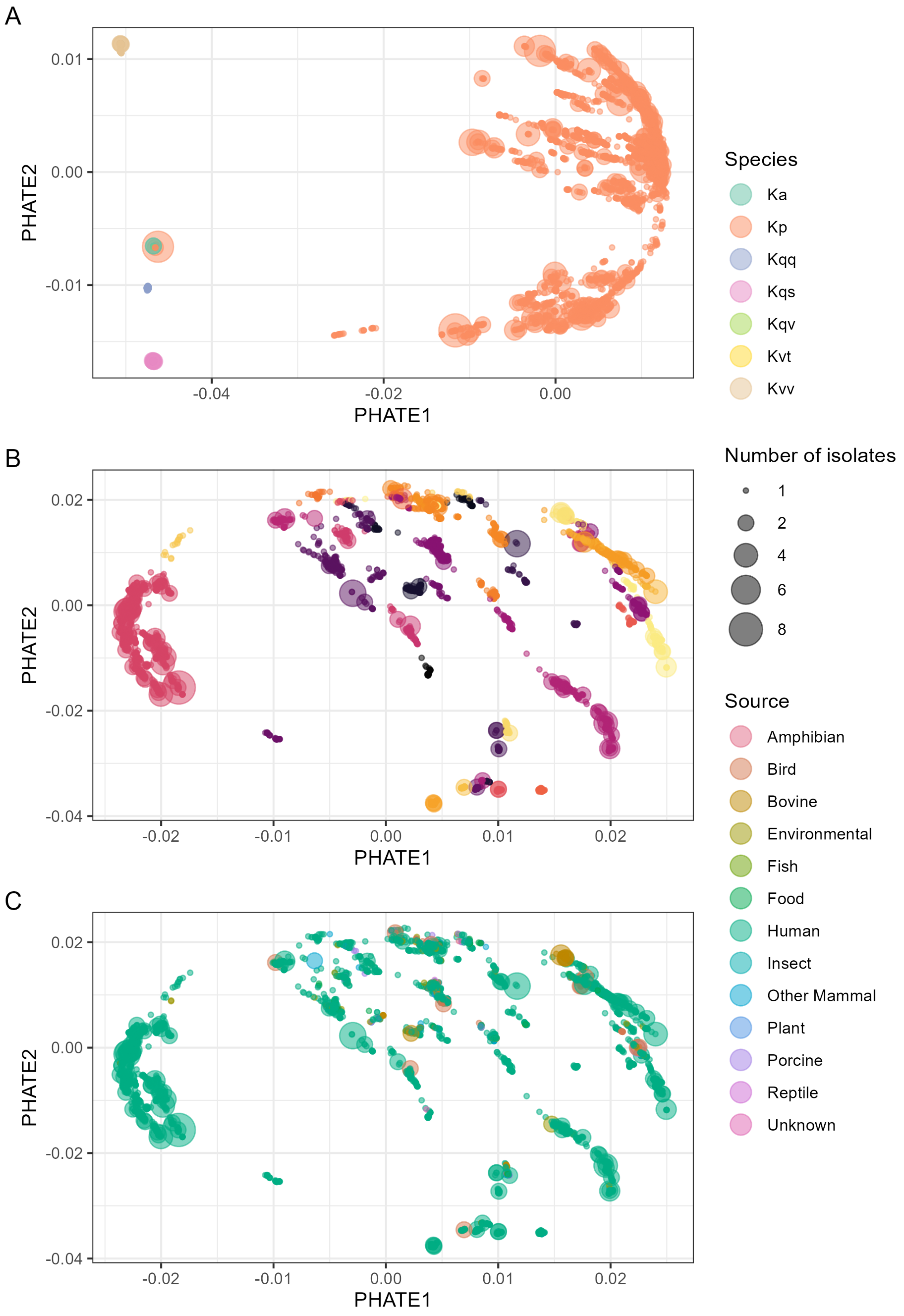

Fig. S4: PHATE analysis of metabolic orthologs.

The first two PHATE dimensions are shown. Colours indicate KpSC taxa (**A**), representatives of the 48 common sublineages (legend not shown for brevity) (**B**) and isolates from different hosts/environmental origins (**C**). The sizes of the points indicate isolate numbers as per the legend. The data underlying this Figure can be found in **S1 and S4 Data**.
