# Supplementary material for: A metabolic atlas of the Klebsiella pneumoniae species complex reveals lineage-specific metabolism and capacity for intra-species co-operation
Source: PLoS Biol. 2025 Dec 12;23(12):e3003559. doi: 10.1371/journal.pbio.3003559 (PMC12700438; doi:10.1371/journal.pbio.3003559)
Supplement: S5 Fig — (PDF) [file pbio.3003559.s014.pdf]

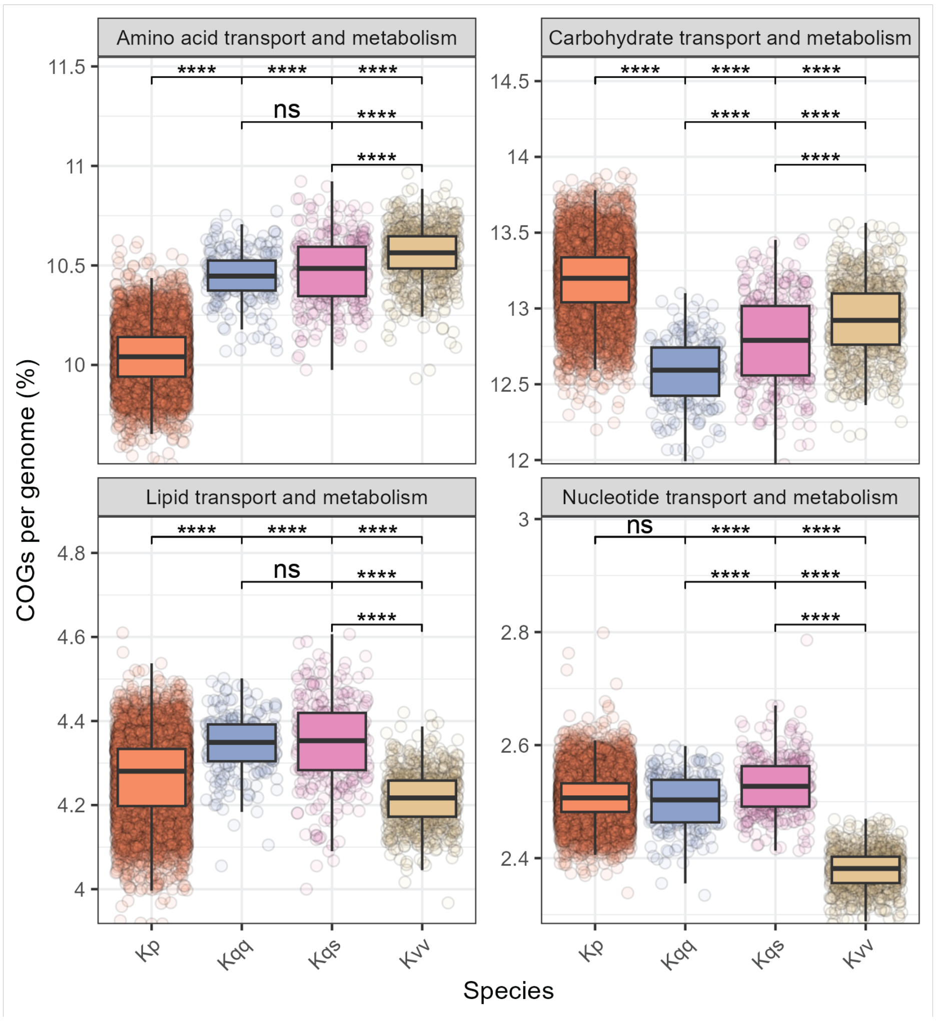

Fig. S5: Taxon-specific specialisation of macromolecular functions

Box and whisker array showing the proportion of each genome assigned to Clusters of Orthologous Gene categories (COG) by taxon coloured as per **Fig. S2**. Dots show individual genomes. Significance is indicated such that the first row shows all comparisons to *K. pneumoniae* (Kp), the second row shows comparisons to *K. quasipneumoniae* subsp. *quasipneumoniae* (Kqq) and third row shows comparisons between *K. quasipneumoniae* subsp. *similipneumoniae* (Kqs) and *K. variicola* subsp. variicola (Kvv). Significance calculated using a non-parametric Kruskal–Wallis test with Holm correction, followed by Dunn's *post-hoc* test (****: $p < 0.0001$, ns: not significant). Only species with n>200 genomes were analysed. The data underlying this Figure can be found at Figshare (https://dx.doi.org/10.6084/m9.figshare.24503737).
