# Supplementary material for: A metabolic atlas of the Klebsiella pneumoniae species complex reveals lineage-specific metabolism and capacity for intra-species co-operation
Source: PLoS Biol. 2025 Dec 12;23(12):e3003559. doi: 10.1371/journal.pbio.3003559 (PMC12700438; doi:10.1371/journal.pbio.3003559)
Supplement: S6 Fig — (PDF) [file pbio.3003559.s015.pdf]

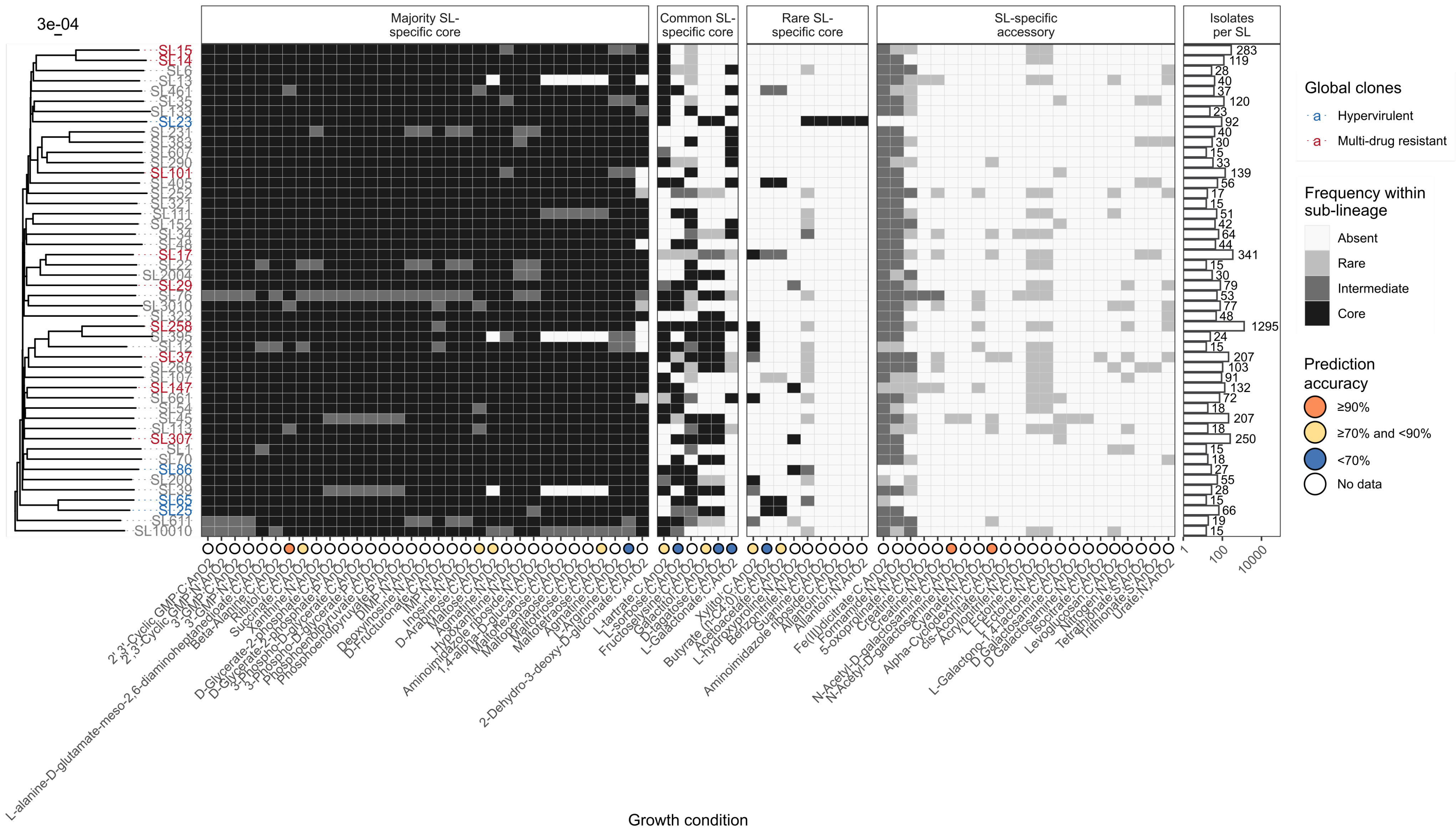

3e-04
Majority SL-specific core
Common SL-specific core
Rare SL-specific core
SL-specific accessory
Isolates per SL
Global clones
Hypervirulent
Multi-drug resistant
Frequency within sub-lineage
Absent
Rare
Intermediate
Core
Prediction accuracy
≥90%
≥70% and <90%
<70%
No data
Growth condition

Fig. S6: Distinct anerobic metabolic fingerprints of sub-lineages within species.

Heatmap showing frequency of variable anaerobic substrate usage across 48 global *K. pneumoniae* sub-lineages (SL). Rows are ordered by phylogeny. Sub-lineage labels are coloured to indicate the globally-distributed clones described in [Wyres KL, Lam MMC, Holt KE. Population genomics of *Klebsiella pneumoniae*. Nature Reviews Microbiology. 2020;18(6):344-59]: Blue shows hypervirulent while red shows multidrug resistant. Substrate shown along X-axis. The element source of each substrate is semi-colon separated and abbreviated for brevity: C = Carbon, N = Nitrogen and S = Sulfur. AnO2 indicates anaerobic conditions. Frequency of substrate usage indicated by shading as shown in legend. Number of isolates per each sub-lineage shown in bars. Tree file available from https://dx.doi.org/10.6084/m9.figshare.24503737. The data underlying this Figure can be found in **S5 Data**.
