# Supplementary material for: A metabolic atlas of the Klebsiella pneumoniae species complex reveals lineage-specific metabolism and capacity for intra-species co-operation
Source: PLoS Biol. 2025 Dec 12;23(12):e3003559. doi: 10.1371/journal.pbio.3003559 (PMC12700438; doi:10.1371/journal.pbio.3003559)
Supplement: S7 Fig — (PDF) [file pbio.3003559.s016.pdf]

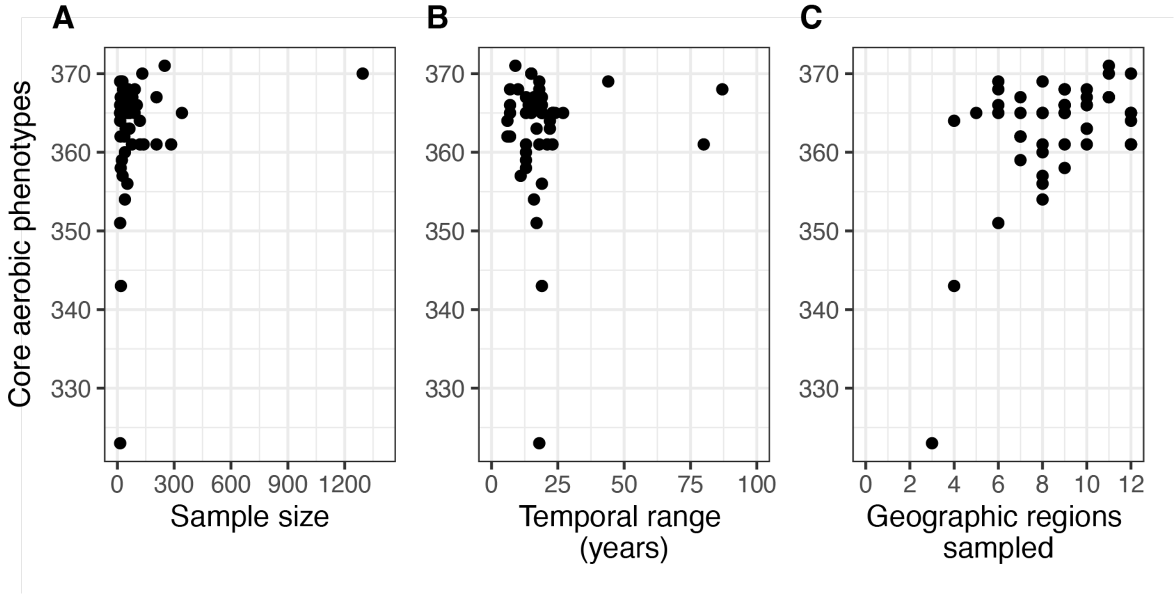

Fig. S7: Relationships between the number of core aerobic phenotypes predicted for each common sub-lineage and sample characteristics.

A: sub-lineage sample size. B: temporal range of collection of isolates represented. C: number of geographic regions represented. The data underlying this Figure can be found in **S1 Data** and **S5 Data**.
