# Supplementary material for: A metabolic atlas of the Klebsiella pneumoniae species complex reveals lineage-specific metabolism and capacity for intra-species co-operation
Source: PLoS Biol. 2025 Dec 12;23(12):e3003559. doi: 10.1371/journal.pbio.3003559 (PMC12700438; doi:10.1371/journal.pbio.3003559)
Supplement: S8 Fig — (PDF) [file pbio.3003559.s017.pdf]

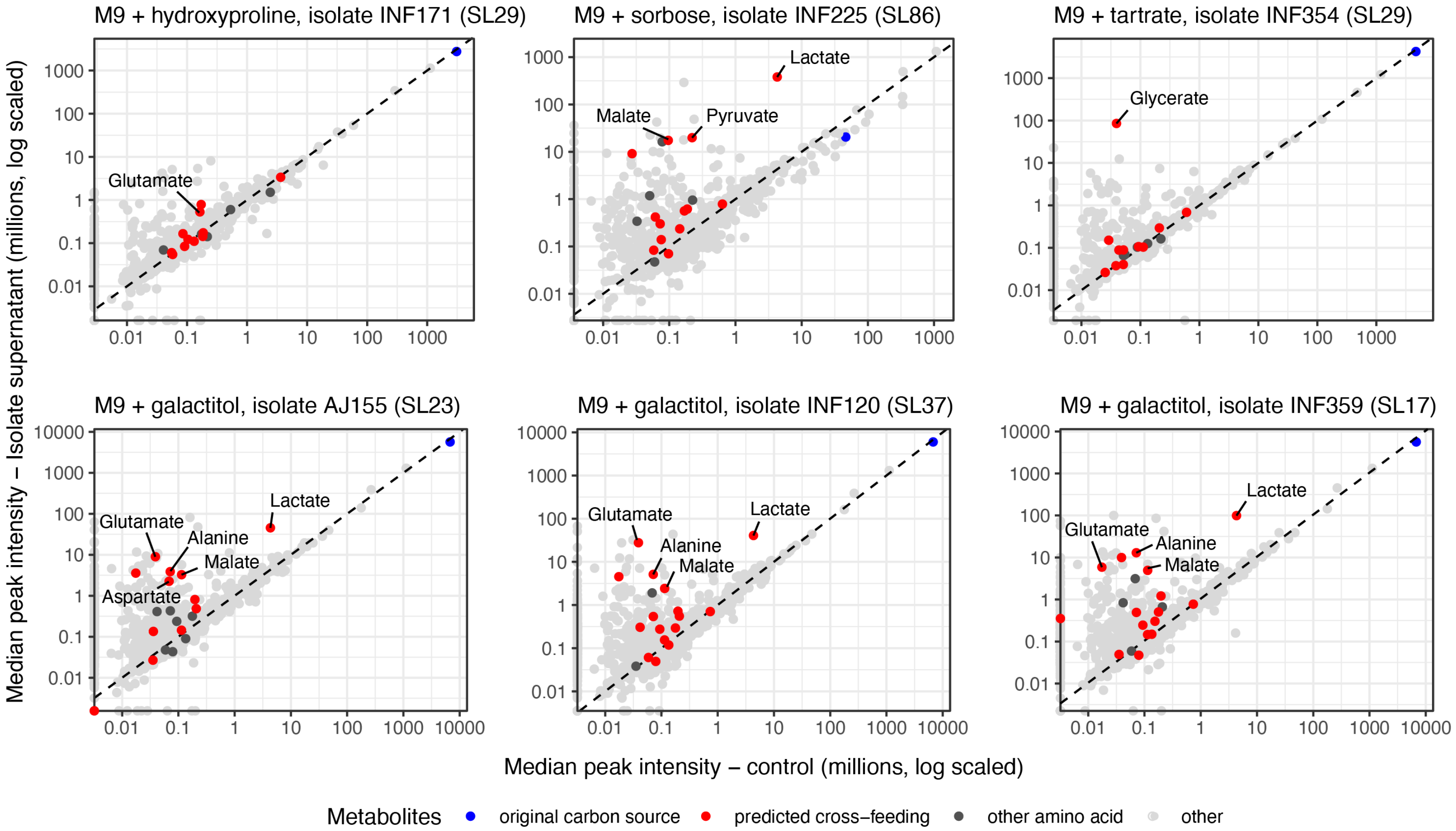

Fig. S8: Metabolite peak intensities in isolate culture supernatants as compared to no isolate controls in M9 minimal media supplemented with various carbon sources as specified.

Points represent median values (four replicates for each isolate, three replicates for each control). Points are coloured as per the legend. The dotted line represents y=x. Labels indicate metabolites with the greatest visual shift from the y=x line, among those predicted to be involved in cross feeding and which are known to support ubiquitous growth of KpSC. The data underlying this Figure can be found in **S6**
