# Supplementary material for: A metabolic atlas of the Klebsiella pneumoniae species complex reveals lineage-specific metabolism and capacity for intra-species co-operation
Source: PLoS Biol. 2025 Dec 12;23(12):e3003559. doi: 10.1371/journal.pbio.3003559 (PMC12700438; doi:10.1371/journal.pbio.3003559)
Supplement: Attachment — Submitted filename: Vezina_metabolic_atlas_rebuttal.pdf [file pbio.3003559.s020.pdf]

Dear Dr Vazquez Hernandez,

We are pleased to submit our revised manuscript for publication in *PLoS Biology*. In line with the Reviewer comments and the Editor's requests, we have substantially reworked the Discussion section and tempered our ecological interpretations throughout. We have clarified the details of the metabolic modelling, including the available accuracy data, which is among the most comprehensive of any multi-strain metabolic modelling analysis published to-date. We have also added new experimental data (metabolomics) to support the cross-feeding experiments. Unfortunately, due to personnel and funding constraints we are unable to perform any further experimental work beyond that now included.

A point-by-point response to the Reviewers' comments is provided below (in blue and indented). We have labelled the reviewer comments by reviewer number and major/minor point numbers to aid referencing. All comments have been addressed.

On behalf of all the coauthors we thank you again for your consideration of this work.

Yours Sincerely,

Dr Ben Vezina and Assoc/Prof Kelly Wyres

EDITOR COMMENTS

In light of the reviews, which you will find at the end of this email, we would like to invite you to revise the work to thoroughly address the reviewers' reports. As you will see below, the reviewers are enthusiastic about the study overall but some suggestions and requests were made that will strengthen the study and shade more light on the mechanism. Reviewer 1 would like more insights into the mechanistic details, e.g. which extracellular metabolites support cross-feeding. The reviewer also gives some suggestions that may improve the models. Reviewer 3 would like you to at least discussed the limitations, including mechanistic insights. However, this reviewer also gives several analytical and experimental suggestions that can enhance the study. Reviewer 4 thinks the study should provide more evidence to support the ecological mechanisms proposed and and the GSMM-derived traits.

We would like to high-light 3 points that should be addressed for further consideration:
1. all four reviewers were concerned that the overall conclusions regarding ecology were too broad and that claims should be toned down and better reflect the data.
2. Additional experiments suggested by the reviewers should be considered, and we strongly encourage that they are performed.
3. Details on the modeling should be provided to allow others to reproduce the results.

> Please see the point-by-point responses below. Line numbers refer to text in the track-changed document.

REVIEWERS' COMMENTS:

Reviewer #1:

This study presents a large-scale metabolic analysis of the Klebsiella pneumoniae species complex. The authors used 7,835 genomes to generate strain-specific genome-scale metabolic models (GEMs) and simulated growth under 1,278 distinct nutrient conditions. They demonstrate that both gene content and predicted metabolic phenotypes are non-randomly structured within the population. Sub-lineages possess distinct "core" metabolic capabilities, some of which enable cross-feeding interactions between lineages. These interactions were validated through in vitro co-culture experiments and in silico simulations. Overall, the study reveals that metabolic differentiation contributes to eco-evolutionary dynamics within KpSC and may inform future therapeutic strategies targeting metabolism. The paper is a really strong and well-executed example of using systems analysis to uncover specific trends to validate. Overall I find the question and approach very timely and exciting.

However, I feel there is very little mechanistic detail to explain their main observation (cross-feeding), and lack of details on the modeling makes it hard for anyone else to reproduce the data.

REV 1 MAJOR POINTS

1. The accuracy and robustness of sub-lineage definitions should be addressed more explicitly. Several sub-lineages appear under-sampled relative to others. Can the authors assess whether these groupings remain robust under alternative clustering methods (e.g., based on gene content or metabolic profiles)? What is the impact of uneven sampling on the core trait designations?

> We would like to clarify that the sub-lineage assignments were not based on any sort of clustering analysis performed as part of this work, but using a standardised, published and stable approach that is based on nucleotide variation in the 629 core genes included in the published KpSC cgMLST scheme (Hennart et al MBE, ref #54). We have amended the Methods section to highlight and further explain these points as shown below. Since our goal was to explore how metabolic diversity is distributed within and between species and sub-lineages, it would be circular to use the metabolic gene or predicted growth phenotype clusters to assign sub-lineages.
>
> **Lines 120-128:** "Pathogenwatch (https://pathogen.watch/) and BIGSdb (https://bigsdb.pasteur.fr/) (51, 52) were used to assign LIN codes (53) and sub-lineages. LIN codes are assigned using core genome MLST profiles that consider variation in 629 core KpSC genes. The third position in the LIN code is used to infer sub-lineage membership (54) and results in groupings that correspond to well-recognised deep-branching monophyletic clades within species. Notably, these sub-lineage assignments do not rely on *de novo* cluster analysis, and are stable with the addition of new genomes."
>
> The reviewer raises an excellent point about the impact of uneven sampling on core trait definitions. We had attempted to limit this impact by application of inclusion criteria for minimum sample size, geographic and temporal representation. We have now also added supplementary plots and statistical tests exploring the impact of these sampling parameters on sub-lineage core trait sets, and a brief discussion at **lines 356-368 as follows:**
>
> "We tested for the impact of sampling biases on our sub-lineage comparisons. The data show no correlation between the number of sub-lineage core traits and either of sample size or temporal range (**Fig. S7A,B**, Pearson's product moment correlation, $r = 0.20$ and 0.04, $p = 0.1751$ and 0.7774, respectively). There was a moderate positive correlation between the number of sub-lineage core traits and number of geographic regions represented (**Fig. S7C**, $r = 0.49$,

p = 0.0004). However, the latter is counter intuitive and seems to be driven by a small number of sub-lineages that have a low number of core traits, rather than a genuine trend (**Fig. S7C**). We are therefore confident that the trends we describe are not the result of sampling biases. Nonetheless, we acknowledge that the precise sets of sub-lineage specific core traits listed here should be considered an approximation, because the addition of further diverse genomes for any given sub-lineage could result in the removal of a minority of traits from its core list."

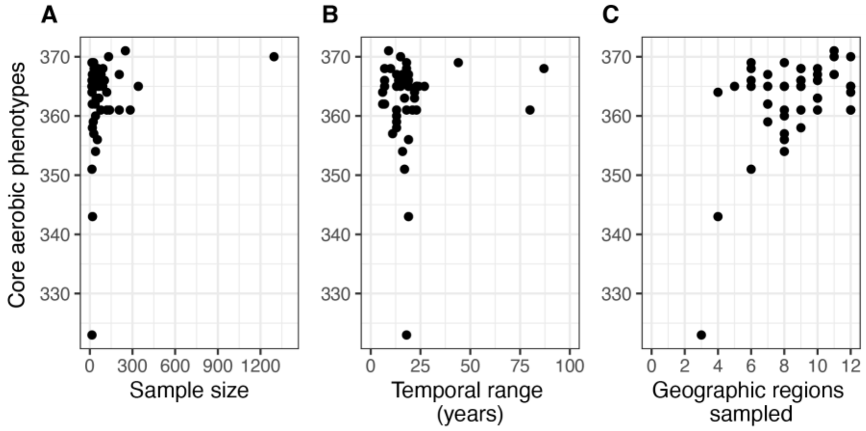

Fig S7: Relationships between the number of core aerobic phenotypes predicted for each common sub-lineage and (**A**) sub-lineage sample size, (**B**) temporal range of collection of isolates represented, (**C**) number of geographic regions represented.

2. The co-culture experiments show convincing evidence of auxotroph growth in the presence of prototrophs, but the mechanistic basis remains speculative. Can the authors predict or validate (e.g., via targeted metabolomics or in silico exchange fluxes) which extracellular metabolites support cross-feeding? Are transporters or known secretion systems encoded in prototroph genomes? Were unrelated sub-lineages or nutrient sources, or heat-killed controls, tested to rule out nonspecific effects? While the focus is on commensalism, did the authors assess whether prototrophs suffer any fitness cost when supporting auxotrophs? This distinction is crucial for determining whether the interaction is commensal, mutualistic, or parasitic. Longitudinal co-culture dynamics or competitive fitness assays would help clarify this.

We have used computational approaches to simulate the interaction of auxotroph-prototroph pairs and predict metabolites involved with cross-feeding. We have also used LC-MS to characterise the metabolites present in the culture supernatants of representative prototrophs, and cross reference with the list of predicted cross-feeding metabolites. In all cases, We were able to identify a significant increase in ≥2 predicted cross-feeding metabolites as compared to no isolate controls. Notably, these include substrates known to

support growth of all KpSC and for which we were able to identify putative exporter genes in the prototroph genomes. The following text has been added to the main manuscript to explain these analyses:

Methods, lines 203-215:
"Metabolomics analysis
We used an untargeted Liquid Chromatography-Mass Spectrometry (LC-MS) approach to explore the metabolites present in the culture supernatant of putative cross-feeding prototrophs. We selected one representative prototroph for each SL-growth substrate combination, and cultured in M9 minimal media plus the carbon source of interest for 18 hours (four biological replicates each, plus three no isolate control replicates for each carbon source). Bacterial cells were pelleted, the supernatant transferred to a new vial and frozen at -80°C prior to transfer to the Monash Proteomics and Metabolomics Platform for LC-MS analysis. Retention times and peak intensities were extracted using IDEOMv21 (70) (see Supplemental Methods for further details). Peak intensities were compared for each prototroph vs the matched no isolate control using a Wilcox Rank Sum test with Benjamini Hochberg multiple testing correction. Missing and zero peak intensity data were imputed as 20% of the minimum peak intensity for each prototroph-control pair."

Results, lines 473-499:
"To further explore these pairwise strain interactions, we performed *in silico* co-culture simulations, which indicated that prototrophs were likely cross-feeding the auxotrophs by catabolising the sole carbon substrates, then exporting metabolites that were capable of supporting auxotroph growth (see Supplemental Results). Subsequent metabolomics analysis of representative prototroph culture supernatants supported the presence of many of the implicated metabolites (**Tables S6 and S8, Fig. S8**): Between two and 13 metabolites predicted to be involved in cross-feeding were detected with significantly higher peak intensity in the prototroph culture supernatant compared to the no isolate controls ($p < 0.05$ by two-tailed Wilcox Rank Sum Test with Benjamini Hochberg multiple testing correction). Among these, between two and 11 metabolites were each detected with >1 log2 fold intensity increase, indicating a substantial increase in the quantity of the metabolite present in the prototroph supernatant as compared to the matched control (**Table S6**). Metabolites with substantial intensity changes included those involved in central carbon metabolism and/or that have been previously shown to support growth for all or almost all KpSC e.g alanine, aspartate, glycerate, glutamate, lactate, malate, pyruvate (68) (labelled in **Fig. S8**).

We cannot rule out the possibility that the putative cross-feeding metabolites were released following cell lysis; however, we note that the associated peak intensity shifts were among the most prominent of all metabolites tested,

including ubiquitous intracellular metabolites e.g. other amino acids (see **Fig. S8**). Therefore it is unlikely that cell lysis alone explains the metabolite quantities we detected. Furthermore, each of the substrates named above is known to be actively exported by the closely related organism *E. coli* (90-95), and a tBLASTn search showed that all six of the representative prototrophs carried orthologs of genes encoding the relevant substrate exporter proteins characterised from *E. coli* (alanine, glutamate, malate, lactate, pyruvate) or *Tetragenococcus halophila* (aspartate). (We were unable to identify any known exporters of glycerate. Also see Supplemental Results.)"

The following information is provided in the Supplementary Materials.

Supplementary Methods, lines 107-141:
"Metabolomics analysis
The following isolates used in co-culture experiments as prototrophs were examined via metabolomic analysis: AJ155, INF359, INF120 (galactitol), INF171 (L-hydroxyproline), INF225 (L-sorbose) and INF354 (L-tartrate) and compared to no isolate controls for each substrate. Briefly, overnight cultures in M9 minimal media + 20 mM glucose were washed twice in 0.9% NaCl, centrifuged at 8,000 x g for 10 minutes. Cultures were then standardised to McFarland Standard 0.45-0.55 in 0.9% NaCl, then inoculated into 5 mL M9 minimal media + substrates (as previously described in co-culture experiments). Performed with four biological replicates except negative controls which were performed in triplicate. Independent cultures were grown aerobically at 37°C, shaking at 200 RPM for 18 hours. Cells were pelleted at 8,000 x g for 10 minutes, then supernatant filtered through a Acrodisc Supor PES Membrane 0.2 µm syringe filter (Pall).

20 µL of cell culture supernatant was treated with 180 µL of ice-cold methanol containing a mixture standard isotope labelled amino acids acting as internal standards. The sample was then mixed at 4°C for 10 minutes on a thermomixer before being subjected to centrifugation at 21,000 x g for 10 minutes at 4°C. 900 µL of the supernatant is then transferred to an LC-MS vial for analysis.

LC-MS analysis of metabolites. Both hydrophilic interaction liquid chromatography (HILIC) coupled to high-resolution mass spectrometry (HRMS). Samples were analyzed on a Vanquish Horizon coupled to Exploris 480 orbitrap mass spectrometer (Thermo Scientific). Chromatography was performed using an iHILIC®-(P) Classic, (PEEK, 150 x 4.6mm, 5µm, 200Å; HILICON, Sweden) with a gradient elution of 20 mM ammonium carbonate (A) and acetonitrile (B) (linear gradient time-%B as follows: 0 min-80%, 15 min-50%, 18 min-5%, 21 min-5%, 24 min-80%, 32 min-80%) at a flow rate of 500 µL/min. The mass spectrometer was operated in polarity switching modes

with a resolution at 120,000. The electrospray conditions were: ionization voltage was 3.5 kV in positive and -2.5 kV in negative mode, ion transfer tube temperature= 325 °C; sheath gas = 60; Aux gas = 15; sweep gas = 2; vaporizer temp = 350 °C.

For accurate metabolite identification, a standard library of approximately 400 metabolites was analysed before sample testing and accurate retention time for each standard was recorded. This standard library also forms the basis of a retention time prediction model used to provide putative identification of metabolites not contained within the standard library (6). Acquired LC-MS/MS data was processed in an untargeted fashion using opensource software IDEOM (7), which initially used msConvert (ProteoWizard) (8) to convert raw LC-MS files to mzXML format and XCMS (9) to pick peaks to convert to.peakML files. Mzmatch was subsequently used for sample alignment and filtering (10)."

Supplementary Results, lines 276-304:
"Identification of putative cross-feeding metabolites
In order to better understand the interactions between the auxotroph and prototroph isolate pairs tested *in vitro*, we simulated co-cultures *in silico* using MICOM for each of one representative pair (10). This approach aims to optimise growth of the community (isolate pairs in this case) and will therefore favour mutualistic interactions. Since we had already demonstrated such interactions *in vitro*, we were seeking to identify the set of putative cross-feeding metabolites, i.e. those associated with reaction fluxes indicating export from the prototroph and uptake by the auxotroph. Between 20 and 29 putative cross-feeding metabolites were predicted for each pair, totalling 44 unique metabolites (**Table S8**). Next we sought to confirm the presence of these metabolites in the supernatants of the representative prototrophs using metabolomics analyses, which were expected to be able to detect 35 of the 44 predicted cross-feeding metabolites (the remaining nine were below the minimum detectable mass, **Table S8**). Notably, 28 of the predicted cross-feeding metabolites were included within the standards library and therefore identified with high confidence. The full list of putative metabolites detected, median peak intensities for the isolate supernatants and matched control samples, log2 fold change and statistical analysis outcomes are listed in **Table S6**.

Several notable predicted cross-feeding metabolites known to support ubiquitous growth of KpSC were associated with substantial peak intensity increases in isolate supernatants compared to the matched controls (labelled in **Fig. S8**). We used a literature search to identify the associated exporter proteins in *E. coli* (alanine, AlaE, NP_417156.1; glutamate, MscS, NP_417399.1; malate, DcuA, CitT, and TtdT, NP_418561.1, NP_415145.1

and NP_417535.1; lactate, SetA, YP_025293.1; pyruvate, PykF, NP_416191.1) or *Tetragenococcus halophila* (aspartate, Asp, BAB92081.1). tBLASTn confirmed that putative orthologs were present in all of the representative prototroph genomes (≥89% coverage and ≥49% identity, with the exception of the aspartate exporter which was found at ~27% identity in all genomes)."

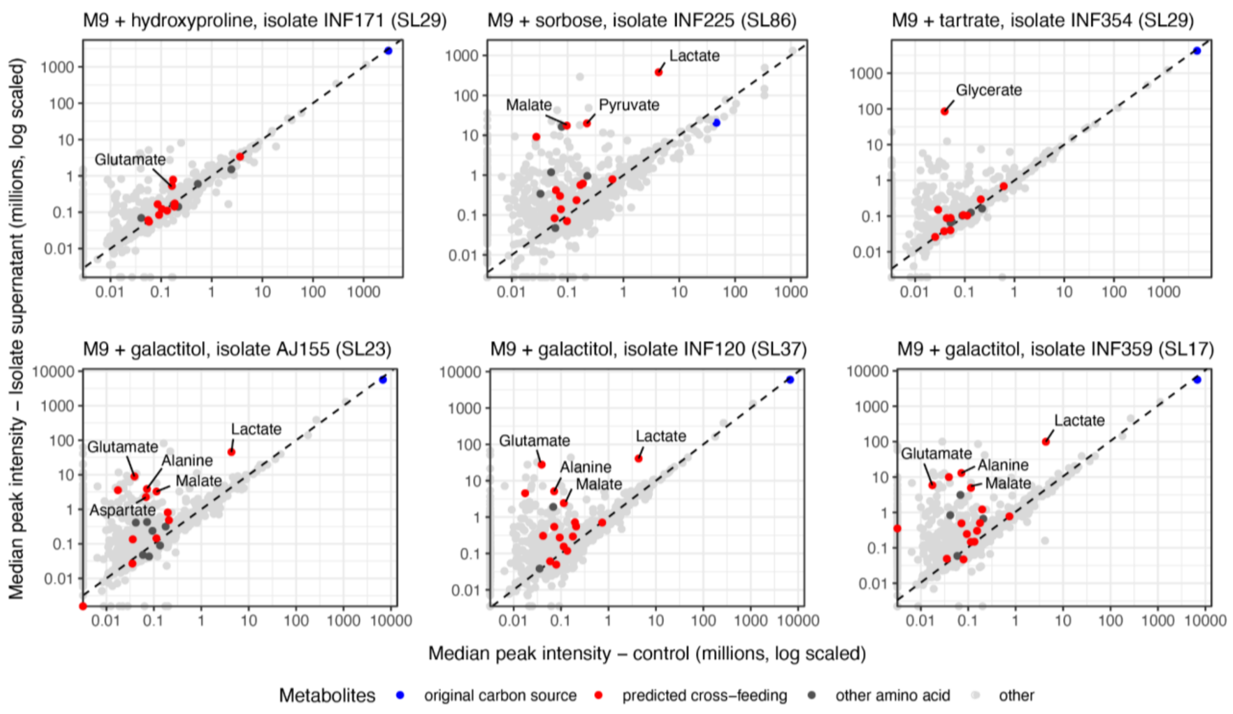

Fig. S8: Metabolite peak intensities in isolate culture supernatants as compared to no isolate controls in M9 minimal media supplemented with various carbon sources as specified. Points represent median values (four replicates for each isolate, three replicates for each control). Points are coloured as per the legend. The dotted line represents y=x. Labels indicate metabolites with the greatest visual shift from the y=x line, among those predicted to be involved in cross feeding and which are known to support ubiquitous growth of KpSC.

3. The manuscript would benefit from greater transparency in strain-specific model construction. Were any manual curation steps performed on the draft GEMs? Can the authors provide SBML, Excel, or JSON versions of representative models (e.g., one per major sub-lineage), including details of media composition and constraints used in flux simulations? Studies like these are always challenging to reproduce since different groups use different standards for flux bound estimates.

We generated draft strain-specific metabolic models using a standardised fully open access and published pipeline (Bactabolize), and open access reference model that were designed to support high-throughput comparative metabolic

modelling studies of *Klebsiella*. (Pipeline available at github.com/kelwyres/Bactabolize and described in ref #63 revised manuscript; reference model available at github.com/kelwyres/KpSC-pan-metabolic-model and described in ref #64 revised manuscript). A subset (2%) of models were gap-filled to enable simulation of growth in glucose which is a ubiquitous phenotype among KpSC. We did not use any additional manual curation because it would not be feasible at this scale. Notably, we have shown previously that our pipeline results in sufficiently accurate draft models for simulating growth phenotypes, including when gap-filling on M9+glucose, without additional curation (notwithstanding all of the recognised caveats of growth prediction, discussed in the manuscript). **We have now deposited all of the final models used in this work in Figshare in json format (https://dx.doi.org/10.6084/m9.figshare.24503737).**

Growth simulations were also conducted using Bactabolize, cycling through each of the carbon, nitrogen, phosphorus and sulphur sources supported by the reference model as sole sources in Bactabolize's default M9 media definition that is freely available as part of the pipeline (https://github.com/kelwyres/Bactabolize/blob/main/data/media_definitions/m9_media.json), and we have now explicitly noted this in the manuscript (as shown below).

The Methods were described in the Supplementary Materials (lines 39-56):

"Metabolic models were generated via Bactabolize v1.0.1 (4) using the *draft_model* command with the following options: '--media_type m9 --atmosphere_type aerobic --min_coverage 25 --min_pident 80'. The *Kp*SC-pan v2.0 model (5) was used as the input reference. One-hundred and sixty of 7,835 models (2.0%) required gap-filling to enable simulation of growth on M9 minimal media with glucose (median: 1 reaction added, range: 1 – 23), using flux balance analysis to optimise the most recent version of the biomass objective function 'BIOMASS_Core_Feb2022'. (Note - we have previously shown that models gap-filled to simulate growth in minimal media plus glucose can accurately predict a range of other substrate growth phenotypes (4).)

Flux balance analysis was used to predict growth phenotypes for all possible carbon, nitrogen, phosphorus and sulfur sources supported by the reference model as sole sources of carbon, nitrogen, phosphorus or sulfur in M9 minimal media defined *in silico*, in both aerobic and anaerobic conditions. These analyses were performed using Bactabolize's *fba* command with the following option: '--fba_spec_name m9'. All growth lower bounds and default substrate sources used in this analysis are available at

https://github.com/kelwyres/Bactabolize. Models were considered capable of simulating growth when the optimised biomass value was ≥0.0001."

For further clarity, we have amended the data and code availability statement in the main text file as follows (lines 224-229):

"All code used to generate metabolic models and simulate growth phenotypes, including media definitions, is available at https://github.com/kelwyres/Bactabolize. The KpSC pan-reference metabolic model is available at https://github.com/kelwyres/KpSC-pan-metabolic-model. All code used to generate figures, simulations and perform statistical analysis can be found at (https://dx.doi.org/10.6084/m9.figshare.24503737), alongside all metabolic models used in analyses."

We therefore believe that this work is highly transparent and reproducible.

4. The low R^2 values for the ANI-Jaccard similarity analyses suggest that metabolic phenotype divergence is not fully explained by core genome similarity. Could the authors provide more context for their interpretation here? Could the extent to which unannotated accessory genes, variation in regulatory architecture, or mobile elements contribute to this unexplained variance? Why else do they expect it to be so low?

We have added a new section to the Discussion that expands this point and connects to the findings from our other analyses (**lines 523-542**):

"Our data shows that metabolic capabilities are structured in the population: we observed significant positive correlations between pairwise ANI and metabolic gene Jaccard similarity among each of the four well-sampled KpSC taxa (Fig. 2a), indicating that closely related strains (high ANI) generally share a greater overlap of metabolic genes than more distantly related strains (low ANI). However, the proportion of variation explained was low (≤0.48 for all taxa). While there is likely a small impact of unavoidable assembly and/or annotation artefacts, we hypothesise the dominant driver is horizontal gene transfer; i) chromosomal recombination, which can introduce nucleotide variation into core genes and inflate ANI values for otherwise closely related genomes that share common metabolic genes; and ii) movement of mobile genetic elements, driving acquisition or loss of genes among closely related genomes (high ANI) and/or acquisition of common sets of genes by distantly related genomes (low ANI). This is further supported by the comparison of pairwise growth prediction Jaccard similarities for isolate pairs within vs between sub-lineages (Fig. 2b, wherein the distributions were overlapping but shifted towards higher values for within sub-lineage pairs) and by our

exploration of 48 common sub-lineages (Fig. 3, Fig. S5). The latter showed that each sub-lineage was associated with a distinct set of core traits (metabolic orthologs and growth phenotypes), alongside clear evidence of variation within sub-lineages, mirroring variations in total gene content that have been reported previously (4, 89)."

We note that these analyses are focussed solely on nucleotide variation and gene content, and do not consider regulation. This caveat is mentioned later in the general discussion about metabolic modelling limitations (**see Reviewer 4, point 2**).

5. While LIN codes offer hierarchical resolution, recombination may erode phylogenetic signal over time. Have the authors considered how homologous recombination affects sub-lineage metabolic coherence and the interpretation of trait inheritance?

Please see response to point 4 just above.

6. The models are stated to have high predictive power, but accuracy varies by substrate, particularly for anaerobic and rare nutrient conditions. Would incorporating confidence scores or ensemble modeling approaches help quantify prediction uncertainty, especially for less-characterized conditions?

The reviewer raises an excellent point, while it is true that the overall model accuracy is very high for the substrates that have been tested, there is also important substrate-specific variation. We have made several text editions to i) clarify when we are referring to overall accuracy and substrate-specific accuracy; ii) highlight that accuracy varies by substrate and clarify the available information. **We have also marked substrate-specific accuracy information in updated Fig 3 and Fig S6.**

Lines 245-251 (Results):
"To analyse the phenotypic diversity across the KpSC, a strain-specific metabolic model was generated for each genome using Bactabolize (63), which was previously shown to produce models with overall 93.2% and 78.1% predictive accuracies in aerobic and anaerobic conditions, respectively (data for 37 diverse KpSC each tested for growth using 124 distinct carbon substrates via the Omnilog microarray; range 5.4-100.0%, median 100.0% accuracy per substrate in aerobic conditions; range 0.0-100.0%, median 88.9% accuracy per substrate in anaerobic conditions (64))."

Lines 393-417 (Results):

“We have previously tested the predictive accuracy of 16 of 68 variable aerobic growth phenotypes, 14 of which were estimated ≥70%, including 12 estimated ≥90% (see Fig. 3), while the accuracy for 11 of 16 tested anaerobic growth phenotypes were estimated ≥70%, including three estimated ≥90% (see Fig. S6). We propose that the majority sub-lineage specific core traits and the accessory traits are unlikely to play a major role in defining sub-lineage specific evolutionary trajectories or ecological interactions, and hence we focussed our follow-up investigations here on the common and rare sub-lineage specific core traits. Our prior work had confirmed predictive accuracies ≥97% for five of the six common sub-lineage-specific core substrates; L-sorbose, L-tartrate, galactitol, D-tagatose and L-galactonate as sole sources of carbon in aerobic conditions (64) (tested using the Omnilog phenotyping microarray, for 37 diverse KpSC isolates). The sixth, fructoselysine, is not present in the Omnilog array and could not be tested here due to its high cost. One of the seven rare sub-lineage specific core substrates, allantoin, was previously tested as a sole source of carbon in aerobic conditions, for which the predictive accuracy was 89.2% (37 isolates tested). Here we tested growth using allantoin as a sole source of nitrogen and found the model accuracy was much lower (15% for 13 isolates tested). We also tested the six other rare sub-lineage specific core growth substrates (13 isolates tested for each), confirming high accuracy (85%) for L-hydroxyproline as a carbon source, 77% accuracy for each of butyrate and methanol as sources of carbon, 46% for xylitol as a source of carbon, 31% for methanesulfonate as source of sulphur and 15% for acetoacetate as a source of carbon (Table S2, see Supplemental Results for more details, Fig. 3).”

Lines 628-643 (Discussion):
“While many of the core substrates and common sub-lineage specific core traits have been confirmed with high accuracy in previous work, we have not tested all substrates and our phenotypic testing in this work showed variable accuracy. This is not unexpected, particularly for rarer substrate usage, which is comparatively understudied and therefore difficult to predict, and/or requires unknown regulatory stimuli. Of note, our assays showed that allantoin nitrogen usage, a trait previously associated with hypervirulent infections (66) and predicted by our models as a sub-lineage specific core trait in the dominant hypervirulent SL23, may in fact be core to KpSC (see Supplemental Results). These and other inaccurate predictions highlight gaps in our current knowledge that are noted for future investigations, and in some cases may have resulted in an overestimation of the population metabolic diversity. On the other hand, given the limitations to modelling traits involving pathways that are not well studied and/or for which the reaction stoichiometries are not defined; and that metabolic models do not consider the impact of sequence variations within metabolic genes nor variations in gene regulation; our

analyses are very likely to represent an underestimate of the true metabolic diversity within and between KpSC species and sub-lineages."

7. Several figures (e.g., Fig. 1C) are difficult to interpret due to the large number of conditions displayed in stacked bar charts. Consider alternative visualizations such as a binary heatmap or dimensionality reduction (e.g., PCA or UMAP) to depict strain-by-substrate growth predictions and their correlation with lineage structure.

We are a little confused here: Fig 1C is the only stacked bar plot in the main manuscript and we are not sure how we might be able to represent Fig 1C as a PCA because it is summarising the diversity of different substrate usages across the whole population, rather than representing variation within/between subgroups. However, we do agree that a PCA or other dimensionality reduction techniques can be useful to visualise how variation is clustered at the species and sub-lineage levels, and have added new **Fig S4** showing a PHATE analysis of the metabolic orthologs.

Fig S4: PHATE analysis of metabolic orthologs. The first two PHATE dimensions are shown. Colours indicate KpSC taxa (A), representatives of the 44 common sublineages (legend not shown for brevity) (B) and isolates from different hosts/environmental origins (C). The sizes of the points indicate isolate numbers as per the legend.

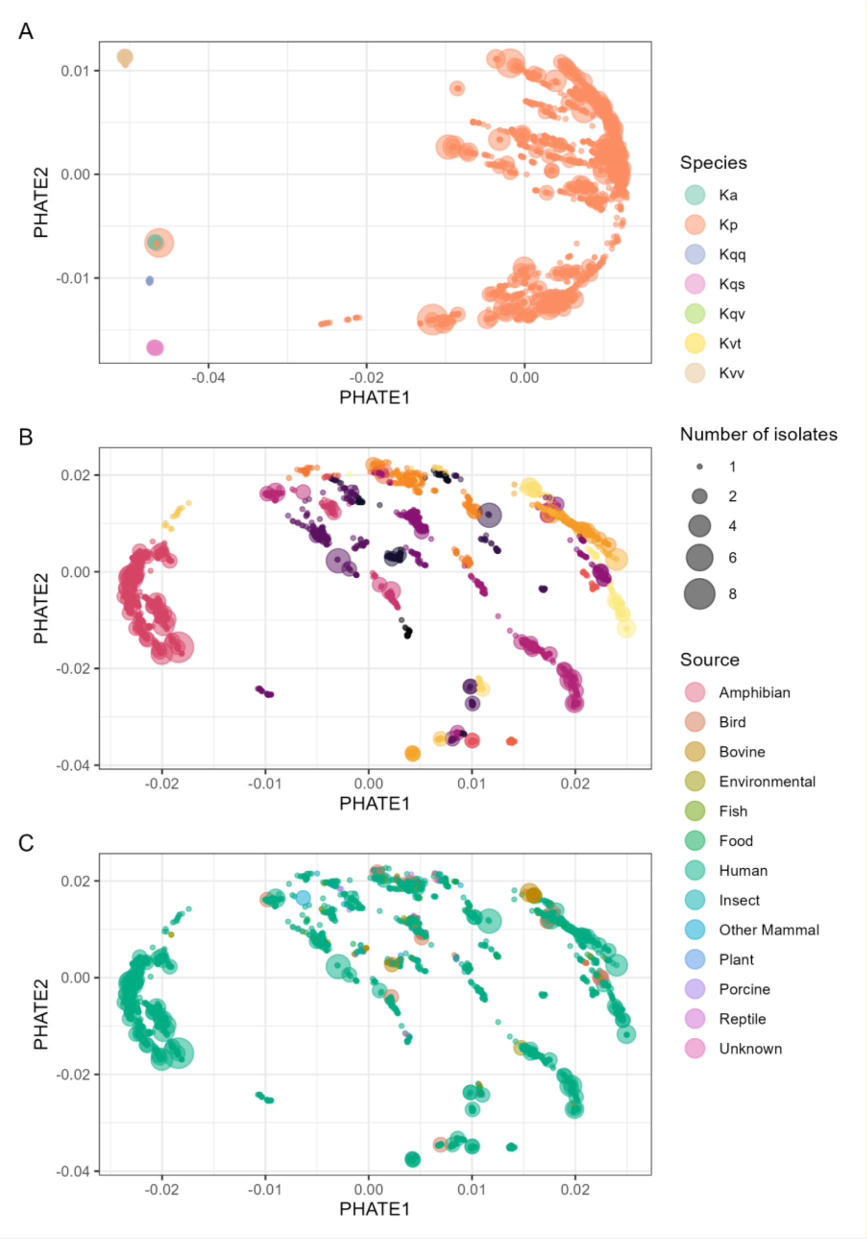

Reviewer #2:

In this manuscript "A metabolic atlas of the Klebsiella pneumoniae species complex reveals lineage-specific metabolism that supports co-existence of diverse lineages" Vezina and co-authors describe an ambitious population-level analysis of over 7000 genomes representing the 7 taxa comprised by the KpSC. The complex harbors

hundreds of diverse sub-lineages with non-random gene content and differential epidemiological behavior. Understanding the processes whereby diversity arises and is maintained in pathogen populations is pivotal for designing disease control interventions. Vezina and co-authors specifically sought to evaluate the distribution and diversity of metabolic traits in the context of the population structure and further aimed to interrogate whether metabolic differentiation underlies the existence and maintenance of co-circulating sub-lineages within the population.

The work combines comparative genomics and quantitative metabolic modeling with a selection of in vitro experiments that yield substantiating empirical evidence for reciprocal cross-feeding between sub-lineages. The scale of the analyses allowed these authors to identify key metabolic differences which appear to indicate metabolic fingerprints on species- and subspecies levels. In my view this is an excellent study. Despite the ambitious scope of this project the authors' description is succinct and quite clear. I accept the rationale behind selections made at successive stages of the analysis. While there are caveats to genome-scale metabolic modeling at this time, the authors are well aware of it. Current limitations are acknowledged in the manuscript and I think they are acceptable. The cooperative co-culture data obtained are technically sound and supportive.

This research makes a very nice contribution. The potential for tailoring Klebsiella pneumoniae control strategies to metabolic processes and dependencies revealed by this study is certainly intriguing. I have no major criticism.

We thank the reviewer for their thoughtful and supportive comments.

Reviewer #3 (Guido Werner):

The study provides a large-scale, systematic analysis of metabolic diversity within the Klebsiella pneumoniae species complex (KpSC). By combining comparative genomics with high-throughput metabolic modeling, the authors introduce new insights into lineage-specific metabolism and its role in bacterial ecology and evolution. The study employs genome-scale metabolic modeling, a powerful computational approach, to predict metabolic traits across 7,835 genomes. The authors validate a subset of predictions using experimental assays, strengthening the credibility of their findings. The study successfully links metabolic variation with phylogenetic structure, demonstrating that metabolic profiles are non-randomly distributed across sub-lineages. This contributes to our understanding of bacterial adaptation and niche specialization. The in vitro co-culture experiments effectively

confirm the existence of reciprocal cross-feeding interactions between sub-lineages, adding empirical
support to the computational predictions. The findings have implications for developing new antimicrobial strategies and microbiome-based interventions, particularly in preventing K. pneumoniae colonization and infection.
I identified some weaknesses/areas for improvement which should at least be considered and discussed in a Limitations paragraph in the Discussion:

REV 3 MAJOR POINTS

1. Incomplete Experimental Validation: While the authors validate some metabolic predictions, only a subset of the proposed lineage-specific metabolic traits are tested. Additional in vitro and in vivo studies would strengthen the conclusions.

> We agree it would be wonderful to validate all of the metabolic traits predicted by the models, however it is simply not feasible to do so, and that is a key reason for utilising the *in silico* modelling approach, rather than relying solely on experimental data.
>
> Our models were previously validated with phenotypic data for n=124 carbon substrates, tested for each of 37 strains using the Biolog microarray platform (aerobic and anaerobic conditions), plus 6 additional substrates that are not found on the Biolog array (Cooper et al Microbial Genomics 2024, ref #64). The Biolog microarray is the gold standard approach for validating substrate usage predictions from metabolic models and interrogation of data for 37 diverse isolates in both aerobic <u>and</u> anaerobic conditions makes our model **one of the most comprehensively tested to-date**.
>
> Nevertheless, we have tested further substrates of interest in this work. We focussed on the aerobic substrate usages that were predicted as common or rare SL-specific core as the most relevant for defining the evolutionary trajectories of sub-lineages (**specified in text lines 426-452**). We were restricted by i) substrate availability and cost (e.g. some substrates cannot be obtained commercially in Australia and/or are prohibitively expensive); ii) availability of physical isolates for testing i.e. we could only test substrates for which we possessed physical isolates predicted to be able to use those substrates. **We have data for all but one common sub-lineage specific core substrate (all with ≥97% accuracy in aerobic conditions) and all rare sub-lineage specific core substrates**.
>
> We have made a number of text changes to enhance the transparency of the available accuracy information and discuss its implications. **Please see response to Reviewer 1, point 6.**

2. Potential Bias in Sample Selection: The dataset primarily consists of human-derived isolates (~83% of the total genomes), which may limit generalizability to environmental and animal-associated strains.

We have expanded the discussion to explain why we believe our results are generalisable to the broader KpSC population (see below) and have added PHATE plots to explore how isolates from different niches cluster by metabolic traits. While the number of isolates from non-human niches is low, our data do suggest that these cluster with their respective sub-lineages i.e. suggesting that the sub-lineage specific relationships are retained. Ongoing work in our team including larger collections of isolates from human gut carriage and non-human niches, (many published after we finalised the dataset for the current manuscript) also supports this trend.

Lines 594-616: "Niche range no doubt further complicates the eco-evolutionary dynamics within Kp populations e.g. where sub-lineages are exposed to fluctuating selective pressures due to transient migration between niches, and/or where sub-lineages undergo niche adaptation. Recent analyses of contemporaneous Kp collections have suggested that some sub-lineages may not be equally distributed between different hosts and environmental sources, which may indicate some level of niche-adaptation (100-102). However, the same studies also identify near-identical strains from multiple sources and a subset of sub-lineages that appear to occur frequently in diverse environments, which is indicative of niche migration. Importantly, the available evidence suggests that wherever KpSC are sampled there exists substantial sub-lineage diversity, and the sub-lineages identified in distinct niches are intermingled throughout the species phylogeny. Therefore, while our convenience sample of genomes comprised primarily human-derived isolates (6,465/7,835 genomes), we anticipate that the trends we report regarding sub-lineage metabolic differentiation are applicable to the broader population. Notably, the limited genomes from non-human niches included here suggest that metabolic traits cluster isolates by sub-lineage rather than by niche (Fig. S4). However, we acknowledge that there may be rarer traits and/or sub-lineages associated with non-human niches that we have not captured in our sample, and studies encompassing more diverse isolates are required to robustly test for niche-metabolism associations."

3. Limited Functional Analysis of Key Metabolic Pathways: While metabolic variations are described at a population level, deeper functional studies on key pathways (e.g., transcriptomics, enzyme activity assays) would enhance mechanistic understanding.

We agree. Our data raise many new hypotheses about KpSC metabolism that we and others can explore- potentially fuelling years of research. However, this is beyond the scope of the current paper.

We had already noted in the discussion that a caveat of metabolic models is that they do not consider regulation. We have now added an explicit statement of the importance of regulation *in vivo* and on the role of other types of data in exploring metabolic diversity **(lines 745-749):**
"Regulatory variations likely play an important role in determining metabolic phenotypes *in vivo*, and in driving competitive interactions and ecological adaptation, and our metabolic models provide a platform on to which transcriptomic, proteomic and metabolomic data can be integrated and contextualised in the future to further probe diversity"

4. Challenges in Interpreting Model Accuracy: The metabolic models rely on databases with inherent gaps in knowledge, leading to potential inaccuracies in predicting rare metabolic traits. The reported discrepancies between predicted and observed phenotypes highlight the need for further refinement.

Please see response to Reviewer 1, point 6.

5. Lack of Direct Clinical Correlations: Although the study discusses potential clinical implications, it does not explicitly analyze the relationship between metabolic traits and clinical outcomes, such as infection severity or treatment resistance.

We are very pleased to learn about the Reviewer's enthusiasm for these sorts of association studies, as we have several such works underway in our team, which will be presented in subsequent publications. Crucially, these studies require very different analytical approaches to those used here, as well as datasets tailored to each of the specific questions under investigation. The aim of the current study was to explore the relationship between metabolism and population structure, which provides essential context with which to interpret any subsequent association studies, and highlights the need for robust statistical analyses that take into account the underlying population structure. **We have added an explicit statement about this to the discussion section (lines 659-663):**
"Our work therefore provides further evidence for the importance of including diverse strains in experimental studies of KpSC competitive interactions, colonisation dynamics, pathogenicity and virulence (including hypervirulence), and highlights the need to consider population structure when designing and interpreting metabolic association studies."

6. Limited study design: While the authors documented a cross-feeding effect across KpSC lineages, the real world scenario includes thousands of commensal

bacteria that may cross-feed KpSC strains across species, genus, family etc barriers under natural conditions, for instance, in the human or animal intestinal tracts.

> The reviewer raises an important caveat of the work that was regrettably omitted from our previous discussion. We have now rectified this, please see **lines 594-569:**
> "We therefore predict that this capacity for 'cross feeding' is a common phenomenon among KpSC, although we cannot comment on its true frequency in natural environments nor in the context of the broader microbial communities within which KpSC exist. Further work leveraging microbial community models in representative conditions, and/or using *in vivo* models will be required to explore these intra-species dynamics."

REV 3 MINOR POINTS

1. Limited Subset of Validated Traits

o Line 351-359 (Page 9): The manuscript states that five of six common sub-lineage-specific core substrates were previously confirmed, but only seven of the rare sub-lineage-specific core growth substrates were tested in this study. It would be beneficial to clarify why these specific substrates were chosen and whether further validation is planned.

o Suggested Improvement: Include a rationale for substrate selection and discuss whether additional phenotypic tests are planned to validate other metabolic traits.

> **Please see response to this Reviewer's major point 1 above, and Reviewer 1, point 6.**

2. Limited Scope of Co-Culture Experiments

o Lines 386-413 (Page 10-11, Figure 4): The co-culture experiments demonstrate cross-feeding between selected sub-lineages, but only a limited number of strain pairs and substrates were tested.

o Suggested Improvement: Explain whether these findings can be generalized across other K. pneumoniae sub-lineages and whether additional co-culture experiments are planned.

> **We have added an expanded discussion section on these points, lines 557-569:**
> "Our co-culture experiments indicated that metabolic variation, including sub-lineage-specific core growth capabilities, can enable co-operation between KpSC wherein an isolate which cannot metabolise a given substrate (an auxotroph), is supported to grow by the breakdown of the substrate by another isolate (a prototroph) (Fig. 4). These findings were replicated for each

of three distinct isolate pairs, representing three distinct and distantly related sub-lineage pair combinations, each tested for two different substrates. In each case, the substrates predicted to support growth of the auxotroph were positively identified within the early log phase culture supernatant of one of the prototrophs, and include those ubiquitously metabolised by KpSC. We therefore predict that this capacity for 'cross feeding' is a common phenomenon among KpSC, although we cannot comment on its true frequency in natural environments nor in the context of the broader microbial communities within which KpSC exist. Further work leveraging microbial community models in representative conditions, and/or using *in vivo* models will be required to explore these intra-species dynamics."

Methodological Clarifications Needed

1. Accuracy of In Silico Predictions

o Lines 220-226 (Page 6, Table S5): The study states that metabolic models were 93.2% accurate for aerobic and 78.1% accurate for anaerobic growth phenotypes.

o Suggested Improvement: Explain the limitations of metabolic models in anaerobic conditions and provide possible strategies for improving accuracy (e.g., integrating transcriptomics or enzyme activity assays).

Please see response to Reviewer 1, point 6.

2. Sample Bias in Genome Selection

o Lines 458-460 (Page 12): The manuscript states that 6,465 out of 7,835 genomes (~83%) are human-derived, potentially limiting ecological generalizability.

o Suggested Improvement: Discuss the potential impact of this bias on the study's conclusions and suggest future studies including more environmental and animal-derived isolates.

Please see response to this Reviewers' major point 2 above.

Lines 251-260: Please add 1 to 2 examples in the main text where genome data support misidentification of Klebsiella species only based on biochemical ID usage patterns.

Unfortunately, we are not aware of any published biochemical profile data with matched genome sequences with which to provide a simple explicit example. Since this is a somewhat tangential point to our key findings, we prefer not to further complicate the work by adding further novel data.

Lines 273-275: Please explain in more details what a low R2 value means and why this indicates a "large amount of unexplained variation".

We have amended the text (shown below) to provide the definition of the R2 value and explicitly state the meaning in the context of our analyses. We have also expanded the discussion on the interpretation of these data (**see response to Reviewer 1, point 4**).

Lines 303-307: "Comparison of the regression gradients indicated significant differences between taxa; however, the R2 values, which indicate how well the models fit the data, were low (range 0.04-0.48), suggesting a large amount of variation in metabolic gene Jaccard similarities was not explained by the relationship with ANI."

Recommendation: Overall, the manuscript presents a well-executed and scientifically valuable study that advances our understanding of K. pneumoniae metabolism and its role in bacterial population structure. Despite some limitations, the findings are compelling and have broad implications for microbial ecology, evolution, and antimicrobial resistance. With minor revisions to address experimental validation gaps and clarify certain methodological aspects, this study should be suitable for publication in a high-impact journal.

We thank the reviewer for their thorough comments, which we have addressed above.

Reviewer #4:

Overall Assessment:
Vezina et al. present an extensive computational analysis of the Klebsiella pneumoniae species complex (KpSC), leveraging pangenomics and genome-scale metabolic modelling (GSMM) across 7,835 genomes, complemented by in vitro experiments. Their central claims are that KpSC sub-lineages exhibit distinct metabolic fingerprints ("sub-lineage-specific core traits") and that observed in vitro cross-feeding indicates metabolic differentiation promotes sub-lineage co-existence via mechanisms like negative frequency-dependent selection (NFDS). While the dataset scale and methodological integration are significant, the study's conclusions suffer from several critical limitations pertaining to model fidelity, interpretation of experimental results, dataset bias, and the inferential leap made regarding ecological mechanisms. The work provides a valuable resource but requires substantial tempering of its core ecological conclusions.
The study reports potentially important findings and a valuable dataset. However, the link between the computational predictions, limited experimental data, and the major ecological conclusions requires significant strengthening and more cautious

interpretation to be suitable for publication in PLOS Biology. Addressing the concerns regarding the robustness of the GSMM-derived traits and the evidence for the proposed ecological mechanisms is critical for publication. I would recommend a revision of the manuscript in this regard.

Please see responses to specific points below.

Critique:

REV 4 MAJOR POINTS

1. Novelty and Significance:
The scale of the comparative GSMM analysis (>7,800 genomes) linked to fine-scale population structure (sub-lineages) is novel and commendable. The study generates a valuable resource (models, phenotype predictions) and presents intriguing findings regarding sub-lineage-specific metabolic potential and in vitro cross-feeding. The novelty primarily lies in the scale of GSMM application to KpSC sub-lineages, rather than the general concept of metabolic niche partitioning in bacteria. The significance of identifying "sub-lineage-specific core traits" is potentially high but is currently undermined by methodological uncertainties (see below). The link established between these traits and ecologically relevant outcomes like stable co-existence is tenuous. The implications for understanding KpSC co-existence and potentially informing control strategies would be highly significant if the robustness of key In Silico findings was established.

We have substantially expanded the description and discussion on the accuracy of the phenotype predictions generated by our models (**see response to Reviewer 1, point 6 and Reviewer 3, major point 1**). We have also added new experimental data supporting the substrates involved in cross-feeding (**see response to Reviewer 1, point 2**). We address additional concerns raised by this Reviewer below.

2. Methodology and Interpretation - GSMM Limitations:
o Sensitivity of "Specific Core Traits": The identification of these differentiating traits is highly sensitive to the inherent limitations of the GSMM approach used. Reliance on incomplete annotations (e.g., KEGG, with ~39% hypothetical genes) means potentially crucial pathways may be missed or mis-assigned. The lack of regulatory networks, kinetics, and accurate prediction for rarer substrates (evidenced by the allantoin discrepancy) severely limits confidence that the predicted "fingerprints" accurately reflect functional differentiation under biologically relevant conditions. A few erroneous predictions could dramatically alter the perceived specificity. Addressing this would require: expanded high-throughput phenotypic validation specifically targeting predicted differentiating traits across multiple isolates

per sub-lineage, sensitivity analyses of the models, and potentially incorporating transcriptomic constraints or probabilistic modelling.

Please see response to Reviewer 1, point 6 and Reviewer 3 major point 1 regarding model accuracy.

We have also expanded the discussion section on the limitations of metabolic modelling and how these may impact the key findings in this work **(lines 621-648):**
"While genome-scale metabolic models are powerful approaches for exploring cellular metabolism, they are not without caveats. Firstly, model construction is reliant on curated databases that are limited by current biochemical knowledge (59), and while our pan-metabolic reference model was built from a large diverse collection of strains (64), it cannot possibly capture all metabolism in the KpSC population. Hence, we are likely underestimating the metabolic diversity within the highly variable KpSC. Secondly, growth prediction accuracies can vary by substrate, and are generally lower for anaerobic conditions for which the metabolic processes are less well understood (64). While many of the core substrates and common sub-lineage specific core traits have been confirmed with high accuracy in previous work, we have not tested all substrates and our phenotypic testing in this work showed variable accuracy. This is not unexpected, particularly for rarer substrate catabolism, which is comparatively understudied and therefore difficult to model, and/or requires unknown regulatory stimuli. Of note, our assays showed that allantoin nitrogen usage, a trait previously associated with hypervirulent infections (66) and predicted by our models as a sub-lineage specific core trait in the dominant hypervirulent SL23, may in fact be core to KpSC (see Supplemental Results). These and other inaccurate predictions highlight gaps in our current knowledge that are noted for future investigations, and in some cases may have resulted in an overestimation of the population metabolic diversity. On the other hand, given the limitations to modelling traits involving pathways that are not well studied and/or for which the reaction stoichiometries are not defined; and that metabolic models do not consider the impact of sequence variations within metabolic genes nor variations in gene regulation; our analyses are very likely to represent an underestimate of the true metabolic diversity within and between KpSC species and sub-lineages. Regulatory variations likely play an important role in determining metabolic phenotypes *in vivo*, and in driving competitive interactions and ecological adaptation, and our metabolic models provide a platform on to which transcriptomic, proteomic and metabolomic data can be integrated and contextualised in the future to further probe diversity."

o Weak Phylogeny-Metabolism Link: The weak correlation reported between core-genome ANI and metabolic gene Jaccard similarity (Fig 2A, low R^2 values) suggests that vertical inheritance within sub-lineages explains only a minor fraction of metabolic potential variation. This challenges the notion of deeply rooted, stable metabolic strategies defining sub-lineages and might point towards more dynamic horizontal gene transfer shaping metabolic capabilities.

Please see response to Reviewer 1, point 4.

3. Methodology and Interpretation - Experimental Limitations:

o Cross-Feeding Extrapolation: Demonstrating increased auxotroph growth in a Transwell co-culture system is interesting but provides insufficient evidence for its role in vivo. The in vitro system lacks host factors, microbiota complexity, spatial structure, and flow dynamics. The biological significance of the observed growth increase is unclear, and whether this specific type of commensalism is a major factor in complex environments is questionable. The selection of specific substrate/strain pairs may also have biased results towards finding interactions.

Please see response to Reviewer 1 point 2, and Reviewer 3, minor point 2.

4. Critique of the NFDS Hypothesis:

o Premature Conclusion: The assertion that observed metabolic differentiation and in vitro commensalism support NFDS as a driving force for co-existence is a significant overstatement based on the data provided. NFDS requires demonstration of frequency-dependent fitness effects (an advantage when rare), which this study does not provide. Commensalism could contribute, but is not sufficient evidence on its own.

o Required Data for NFDS: Substantiating metabolically-driven NFDS requires substantially different data, such as: (i) Longitudinal studies tracking sub-lineage frequencies and resource availability in a shared environment; (ii) Experimental evolution demonstrating stable co-existence or frequency oscillations linked to metabolic capabilities; (iii) Direct competition assays showing an inverse relationship between frequency and fitness under relevant limiting conditions; (iv) In situ functional genomics/metabolomics linking pathway activity to abundance; (v) Population genomic analyses seeking signatures of balancing selection on the differentiating metabolic genes.

We have made a number of text edits to contextualise and temper our claims about NFDS.

We have changed the title as follows: "A metabolic atlas of the *Klebsiella pneumoniae* species complex reveals lineage-specific metabolism and capacity for intra-species co-operation."

We have edited the abstract to remove mention of NFDS and temper the claims on the relevance of our findings to the co-existence of sub-lineages e.g. **lines 30-33:**
"A substantial fraction of the pan-genome is predicted to be involved in metabolic functions and hence these data are consistent with metabolic differentiation at the sub-lineage level."

And lines 43-46:
"We propose that these substrate specialisations may promote the existence and persistence of co-circulating sub-lineages by reducing nutrient competition and facilitating commensal interactions."

We have edited the introduction, lines 72-74:
"We therefore hypothesised that sub-lineages differ in terms of metabolic capabilities, which likely contributes to their epidemiological behaviours and may facilitate the maintenance of diverse sub-lineages within the population."

And lines 103-105:
"including multi-drug resistant and hypervirulent clones, are associated with distinct substrate usage profiles that appear to facilitate commensal interactions and may promote co-existence."

We have reworked the discussion section on this point, lines 571-592:
"Intriguingly, our data may shed light on the question of how so many sub-lineages can be maintained within the KpSC population. In the most intuitive scenario, the ability to consume different substrates may enable KpSC sub-lineages to inhabit distinct metabolic niches within spatially overlapping environments, thereby reducing the impact of nutrient competition. Supporting this hypothesis, it has been shown previously that this type of metabolic niche differentiation can support the co-existence of multiple strains of *E. coli* in an *in vivo* model of mouse gut colonisation (97). In a more complex scenario, the ability to consume a substrate that cannot be consumed by a competitor can provide a selective advantage resulting in a relative frequency increase. This in turn can result in a depletion of the food resource and shift in selective pressure to favour the competitor. Although comparatively understudied, commensal interactions such as the metabolite cross-feeding implicated by our co-culture experiments (Fig. 4), can also drive similar frequency-dependent selective shifts (99). Over time, such fluctuations may prevent the exclusion of sub-lineages and facilitate a stable equilibrium of co-existence. Notably, a similar model of negative frequency-dependent selection has been proposed previously as a driver for the maintenance of diverse sub-lineages (entities) within bacterial populations (98, 99). However, this model is notoriously difficult to substantiate (99), requiring a long-term dataset from the

same location over time and/or laboratory protocols that are beyond the scope of the current work, and therefore it remains to be confirmed."

5. Ecological Relevance and Dataset Bias:

o Clinical Skew: The overwhelming bias towards human clinical isolates (~82%) severely restricts the generalizability of the findings. The identified metabolic patterns likely reflect adaptations to human host niches and infection pressures, not necessarily the broader ecological strategies of KpSC across its diverse habitats (environmental, non-human hosts).

We disagree. **Please see response to Reviewer 3, major point 2.**

o Host/Niche Context: The study lacks integration of host metabolic context or interaction with other microbiota members. Fitness in vivo is shaped by host nutrient provision, immune factors, and competition/cooperation within complex microbial communities. Extrapolating from minimal media simulations and simple in vitro tests to these complex scenarios is problematic. Addressing this requires: Niche-stratified sampling and comparative analysis, context-specific modelling using media mimicking in vivo conditions, integration with host/metagenomic cohort data, and validation in relevant in vivo or complex ex vivo models.

See response to Reviewer 3, minor point 2.

6. Technical Soundness: The core computational pipelines appears technically sound. However, significant concerns arise regarding the interpretation and robustness of conclusions derived from the GSMMs due to acknowledged (but perhaps underemphasized) limitations like annotation gaps, predictive inaccuracies for key differentiating traits, and the absence of regulatory dynamics. Furthermore, the link drawn between the in vitro cross-feeding results and the complex ecological mechanism of negative frequency-dependent selection (NFDS) is currently speculative and lacks sufficient direct evidence. The strong bias towards clinical isolates also limits the generalizability of the ecological conclusions.

Please see the responses to Reviewer 1, point 6; Reviewer 3 major points 3 and 6; Reviewer 3, minor point 2.

Required Revisions

Major revisions are necessary to address these points and strengthen the manuscript.

Strengthen Evidence for Ecological Mechanisms: The claims regarding NFDS must be substantially tempered or supported by more direct evidence. This might involve new analyses (if possible with existing data, e.g., population genomic scans for balancing selection on key metabolic genes) or, more likely, a significant reframing of the discussion to present NFDS as a hypothesis suggested by, but not proven by,

the current data. Explicitly discussing alternative ecological models would also be beneficial.

Please see responses above for this reviewer.

1. Address GSMM Limitations & Validate Key Predictions: The authors need to more critically assess and discuss how model inaccuracies and limitations impact the identification and interpretation of "sub-lineage-specific core traits." Ideally, expanded experimental validation focusing specifically on these predicted differentiating traits across more isolates/conditions, or computational sensitivity analyses, would be needed to bolster confidence in these core findings.

Please see responses above for this reviewer.

2. Contextualize Findings Regarding Data Bias: A more nuanced discussion is required regarding how the clinical isolate bias likely shapes the observed metabolic landscape and limits extrapolation to other environments. Explicitly framing the findings in the context of adaptation to human/clinical niches would be more accurate.

Please see responses above for this reviewer.

3. Refine Interpretation: Overall, the manuscript needs careful revision to ensure conclusions, particularly ecological ones, do not overreach the presented data.

Please see responses above for this reviewer.
